# Supplementary material for: Detection of elusive DNA copy-number variations in hereditary disease and cancer through the use of noncoding and off-target sequencing reads
Source: Am J Hum Genet. 2024 Mar 25;111(4):701–13. doi: 10.1016/j.ajhg.2024.03.001 (PMC11023916; doi:10.1016/j.ajhg.2024.03.001)
Supplement: Document S1. Figures S1–S7 and supplemental methods [file mmc1.pdf]

The American Journal of Human Genetics, Volume *111*

Supplemental information

Detection of elusive DNA copy-number variations in hereditary disease and cancer through the use of noncoding and off-target sequencing reads

Mathieu Quinodoz, Karolina Kaminska, Francesca Cancellieri, Ji Hoon Han, Virginie G. Peter, Elifnaz Celik, Lucas Janeschitz-Kriegl, Nils Schärer, Daniela Hauenstein, Bence György, Giacomo Calzetti, Vincent Hahaut, Sónia Custódio, Ana Cristina Sousa, Yuko Wada, Yusuke Murakami, Almudena Avila Fernández, Cristina Rodilla Hernández, Pablo Minguez, Carmen Ayuso, Koji M. Nishiguchi, Cristina Santos, Luisa Coutinho Santos, Viet H. Tran, Veronika Vaclavik, Hendrik P.N. Scholl, and Carlo Rivolta

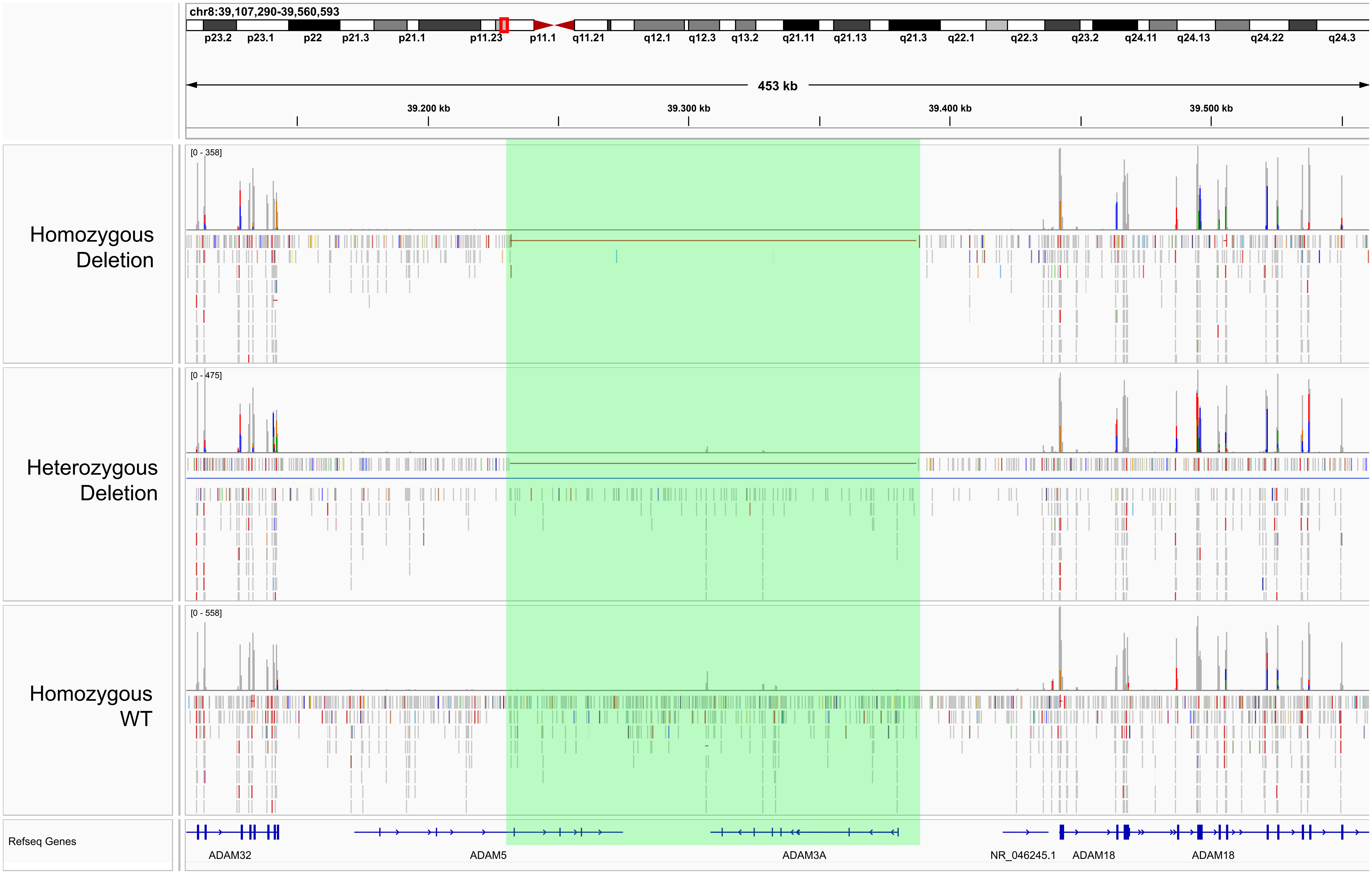

Figure S1: IGV view of a typical deletion involving mostly off-target regions. A common deletion on chromosome 8 is shown (gnomAD: DEL_8_91589, in green). Sequencing data presented here are derived from (top to bottom): a homozygous, a heterozygous, and a wild type (WT) individual for the deletion. Split reads are shown as red horizontal lines.

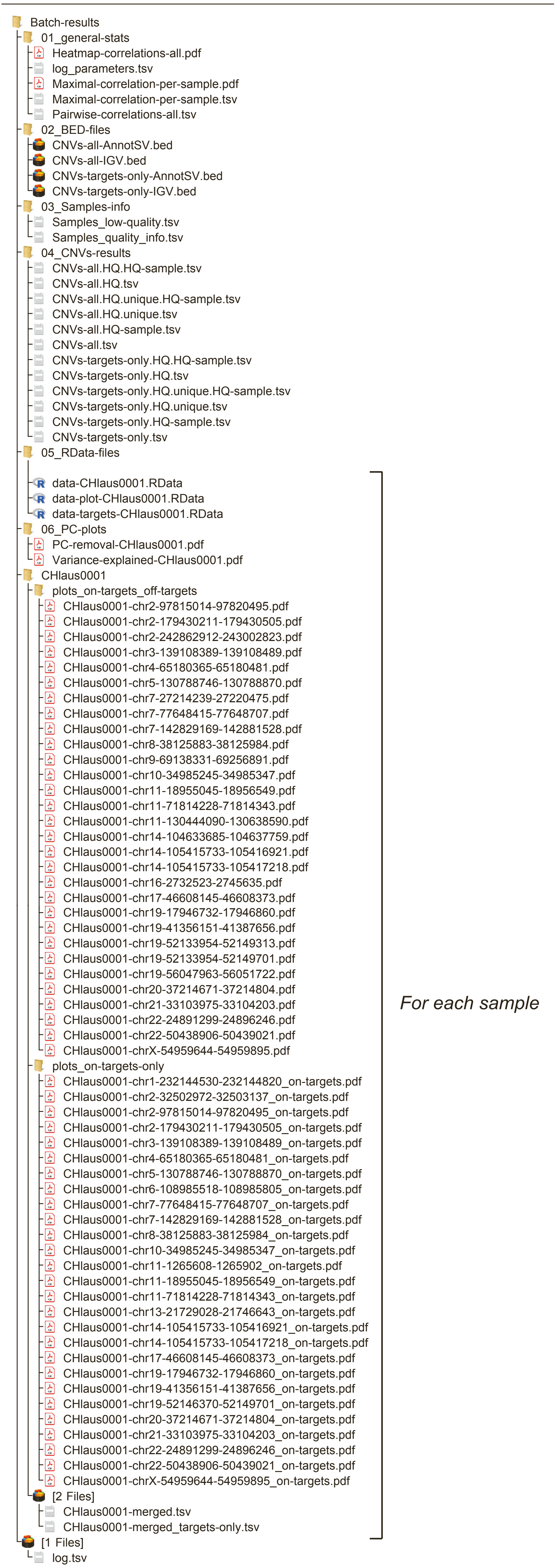

Figure S2: Typical output files of OFF-PEAK, here for individual CHlaus0001. The files listed at the top are common to all samples belonging to the same batch, whereas those listed at the bottom are specific for each sample.

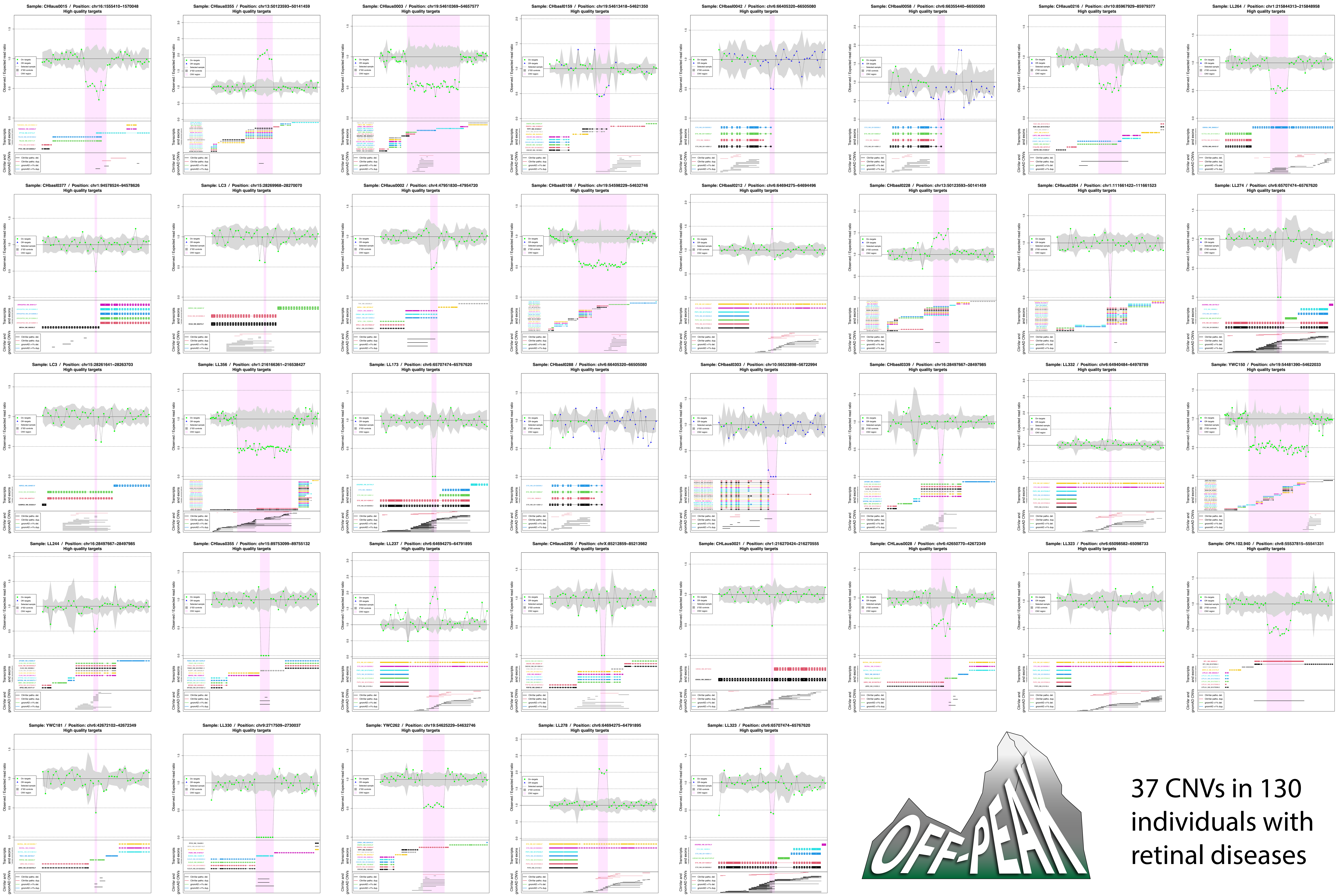

Figure S3: OFF-PEAK graphical outputs for all validated CNVs, detected in the retinal diseases cohort.

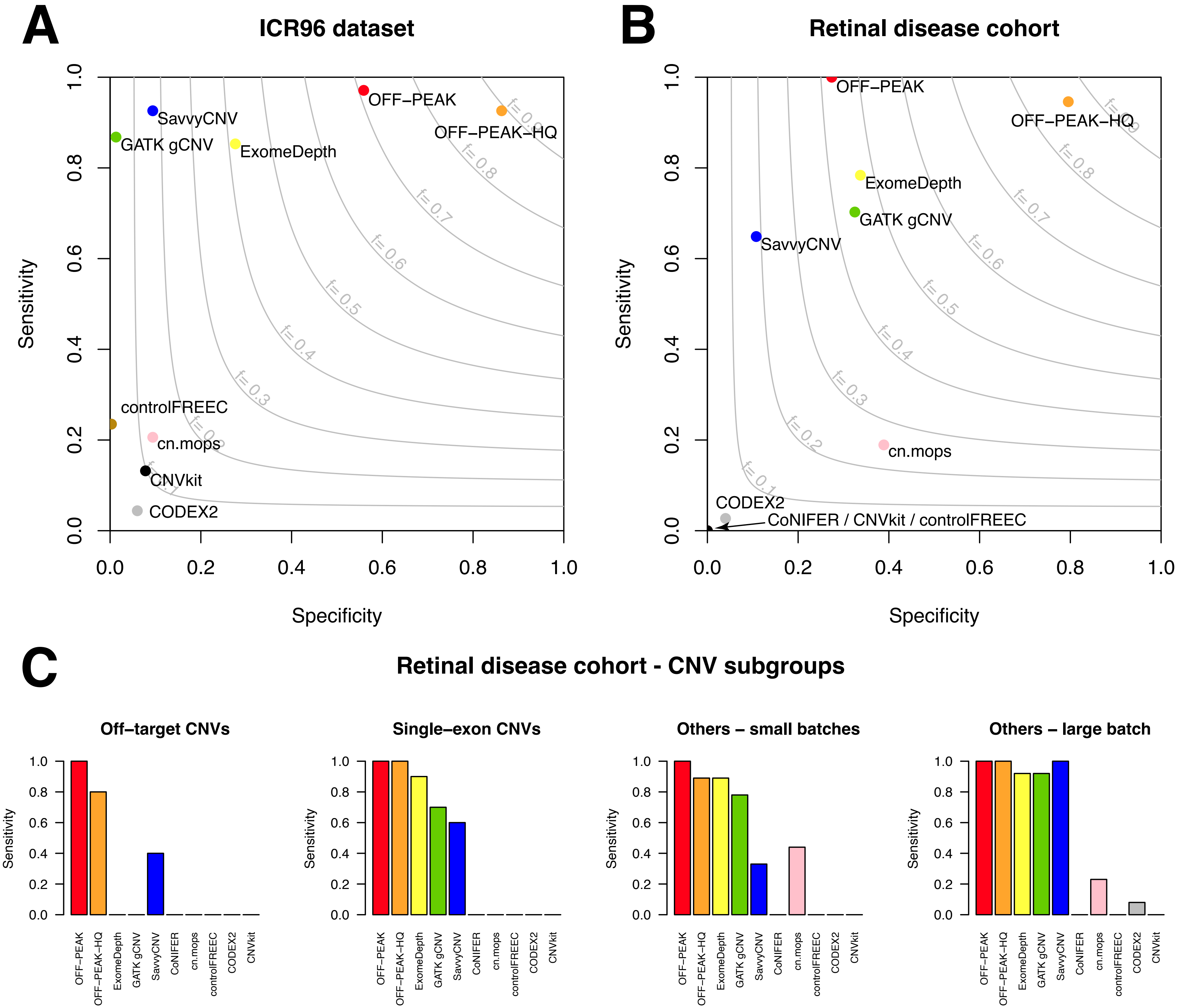

Figure S4: Performance of OFF-PEAK and other tools with respect to different testing sets, taking only CNVs with exact captured regions detected. (A) Specificity-sensitivity plot for the ICR96 dataset of 96 cancer samples, on 68 validated CNVs. (B) Specificity-sensitivity plot for the cohort of 130 individuals with retinal phenotypes, on 37 validated CNVs. (C) Bar plots of sensitivities for the latter cohort, stratified according to the type of CNVs considered: off-target (e.g. noncoding), on-target single-exon, small batches (kits 1 and 3), and large batch (kit 2, see Sup. Methods). The curves in light grey represent the F-score or harmonic mean of sensitivity and specificity.

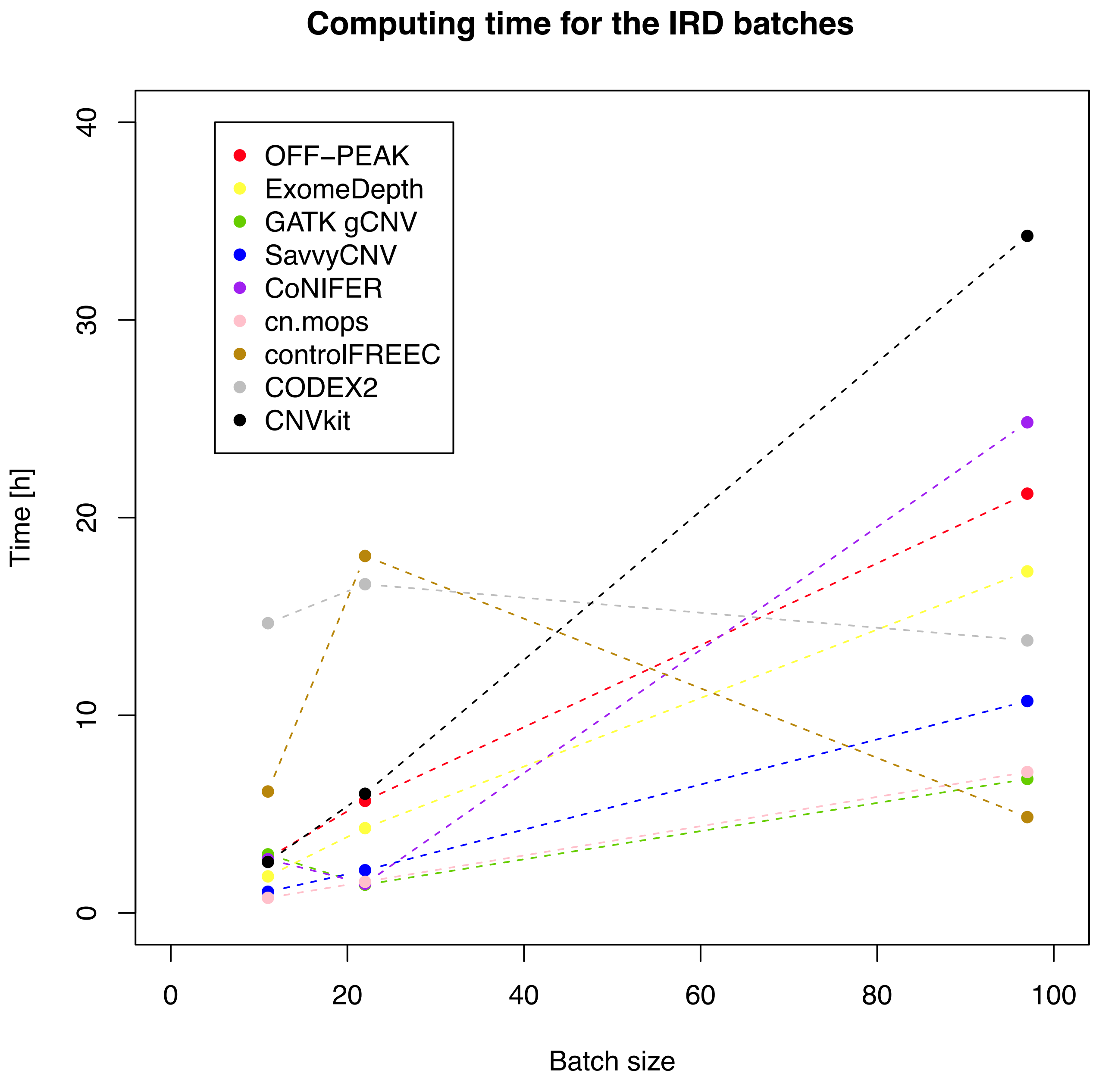

Figure S5: Computing time for three WES batches of various sizes (kit 1, n =11; kit 2, n=97; kit 3, n = 22).

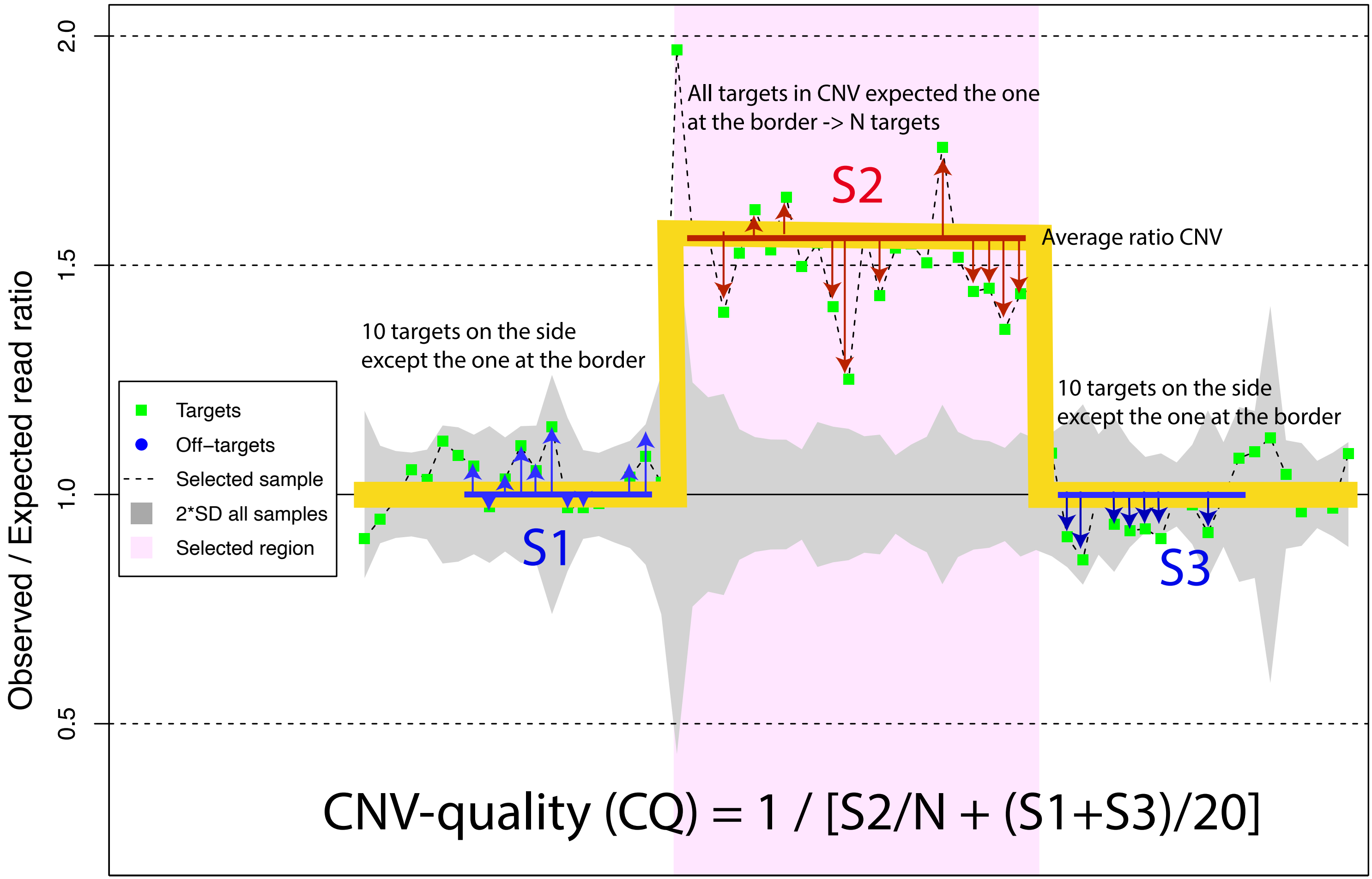

Figure S6: Schematic view of CNV-quality (CQ) computation. A CNV determines an abrupt change in coverage, which can be represented by a rectangular function (in yellow). We can estimate the quality of a CNV by computing how close such variation of coverage and this rectangular function are. For this purpose, we sum the deviations in coverage of the targets (green squares) from this function. Deviations occurring outside of the CNV are highlighted by blue arrows, whereas red arrows highlight those occurring within the CNV. The exact equation estimating CNV quality is indicated at the bottom of the image.

A

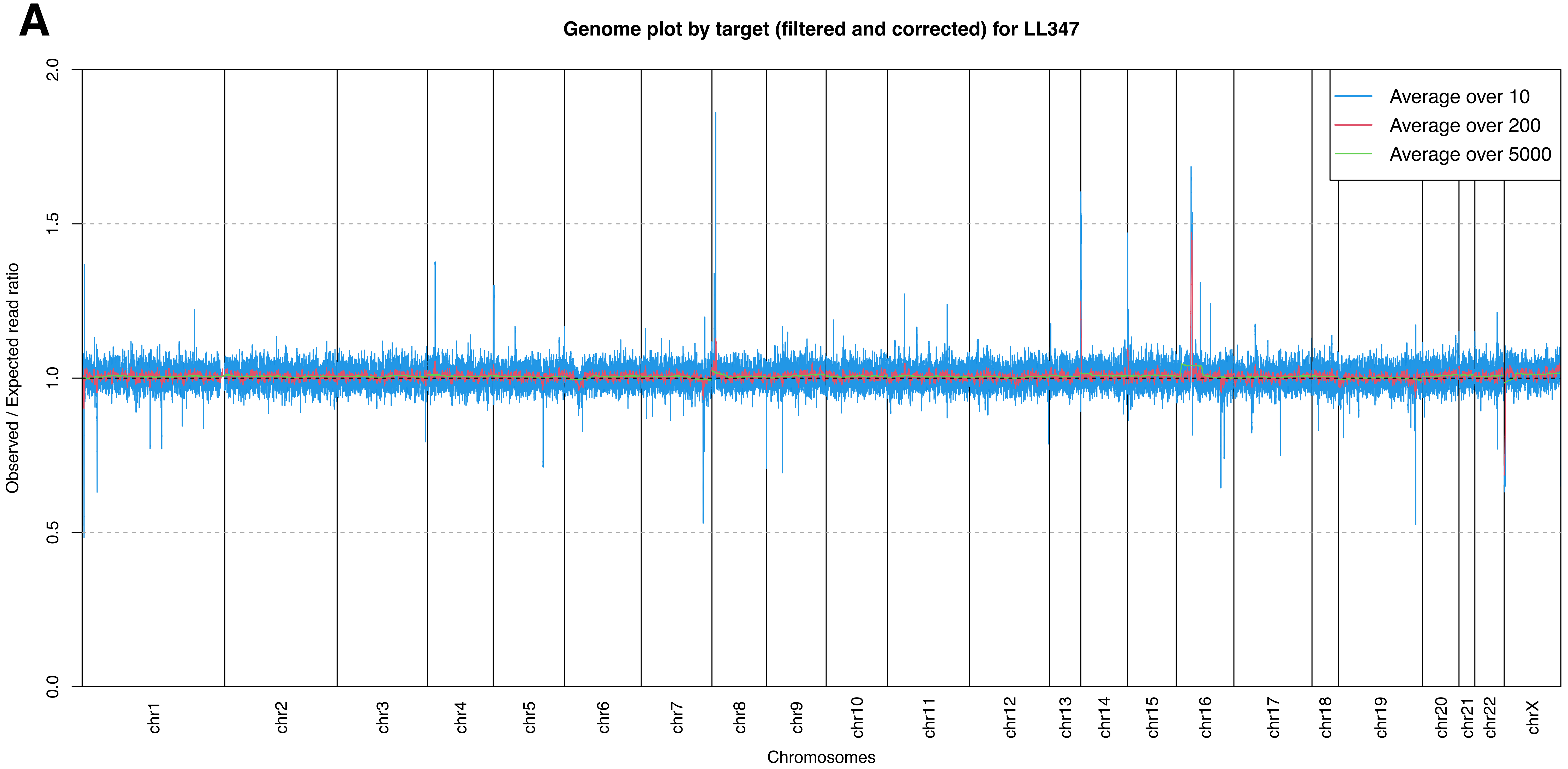

B

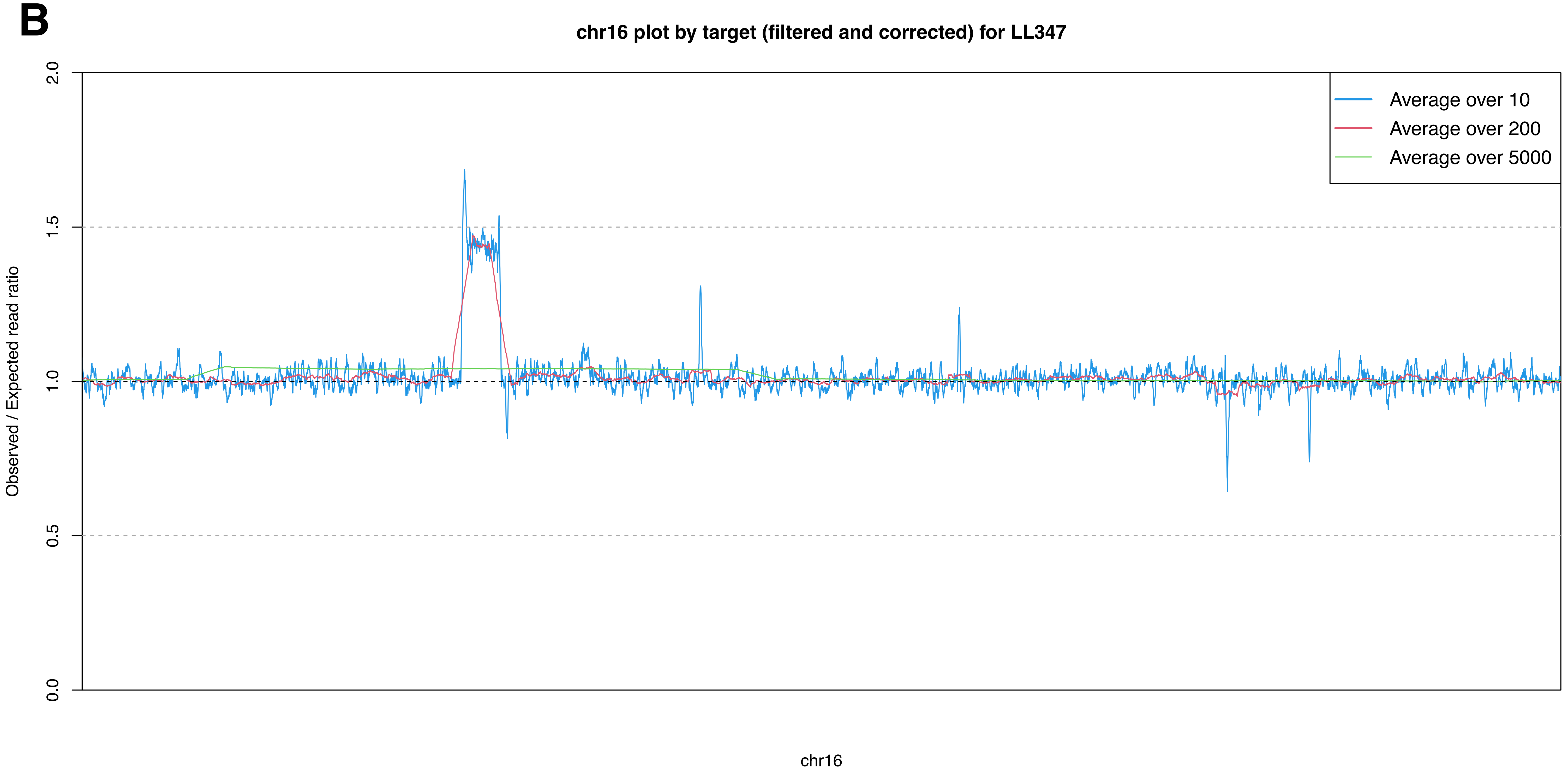

Figure S7: Examples of a genome-wide coverage plot (A) and a chromosome-specific coverage plot (B) for individual LL347, who carries a large heterozygous duplication on chromosome 16.

Supplementary Methods

Details about noise removal using LOO-PCA

Noise removal was done using a leave-one-out principal components analysis (LOO-PCA) approach. Specifically, the optimized number of PCs to be removed was computed by:

1. Downsampling of the targets, by randomly selecting a specific number of them (--downsample, default: 20,000).
2. Creation of artificial (fake) heterozygous deletions and duplications by halving or multiplying by 1.5 times the real coverage value of a defined number of regions in the test sample (--nbFake, default: 500 each).
3. Computing PCA on the control samples only (LOO-PCA).
4. Starting to recover CNVs without PC removal, then incrementing the removal of PCs and computing at every step the AUC based on Z-scores of the test sample compared to control samples. The optimized PC removal was defined when the performance increased by less than a specific threshold (--stopPC, default: 0.0001). If the threshold was passed, 10 more PCs were sequentially removed to try to increase the performance.

CNV annotation and graphical representation by OFF-PEAK

Following the detection process, all CNVs were annotated to include: the sample they belonged to, their genomic coordinates (minimal and maximal), their type (deletion or duplication), their reads ratio compared to controls, Z-score, ploidy, affected targets, affected

exons, genes, non-coding RNAs and functional elements affected (RefSeq), the number of samples with overlapping CNVs, the overlapping CNVs from ClinVar and gnomAD databases, as well as with various quality metrics.

The quality metrics were:

- PQ (ploidy quality) = minimal difference of Z-score between the called ploidy and other ploidies divided by number of targets
- CQ (CNV quality) = measure of CNV quality based on divergence from a rectangular function (difference between expected and observed ratios), defined as:

$$CQ = \frac{1}{\frac{S2}{N} + \frac{S1+S3}{20}}$$

for a CNV going from target *k* to *m*:

$$S1 = abs\left[\sum_{k-11}^{k-2} (x_i - 1)\right]$$

$$S2 = abs\left[\sum_{k+1}^{m-1} \left(x_i - \frac{\sum_{i=k+1}^{m-1} x_i}{m-k-2}\right)\right]$$

$$S3 = abs\left[\sum_{m+2}^{m+11} (x_i - 1)\right]$$

(see Figure S6)

- QUAL = Z-score * PQ * CQ / N-targets. It measures both, the quality of the called ploidy (PQ), and that of the correct CNV “shape” (CQ)

The graphical representation of every CNV detected shows the ratio between the sample considered and the control samples, the genes and exons involved, as well as frequent CNVs

from gnomAD and pathogenic CNVs from ClinVar. The canonical transcripts were indicated based on NCBI RefSeq Select.

Other output files by OFF-PEAK

These additional output files were created when running OFF-PEAK:

- Detected CNVs with annotations
- CNV plots for top 20 CNVs per sample, including CNVs found in ClinVar and gnomAD databases (examples in Figure 5 and Figure S3)
- Heatmap of the correlation between samples and the table of pairwise correlations
- Bar chart of the maximal pairwise correlation per sample, as well as the data in text format
- BED file compatible with the AnnotSV software[1]
- BED file for the Integrative Genomics Viewer[2]
- Sample information, such as quality or number of detected CNVs
- RData files for creating additional plots
- Plot showing performance of PC removal on artificial CNVs and plot showing the cumulative explained variance
- Genome and chromosome plots (example in Figure S7)

Parameters used for CNV detection on the ICR96 data

ICR96 data (FASTQ files and target BED file)[3] were downloaded from the European Genome-phenome Archive (EGA), with the agreement of Prof. Nazneen Rahman at The

Institute of Cancer Research, London. The FASTQ files were used as described in the "Mapping and variant calling" section to produce processed BAM files. CNV detection was run as follows:

- OFF-PEAK: default parameters with target BED file
- cn.mops: default parameters
- CNVkit: default parameters with target BED file
- CODEX2: default parameters with target BED file, without chromosome X
- CoNIFER: did not work due to insufficient number of target regions ("Error: This chromosome has fewer informative probes than there are samples in the analysis!")
- ExomeDepth: default parameters
- Control-FREEC: default parameters
- GATK: default parameters with target BED file
- SavvyCNV: default parameters; after running CoverageOffTarget command, SavvyCNV was run with -d 200

Data processing for the validation on the ICR96 dataset

We retrieved the outputs of each tool and processed them as follow:

- OFF-PEAK: all CNVs detected (deletion if ratio < 1 and duplication if ratio > 1) affecting targets (CNVs-targets-only.tsv file)
- OFF-PEAK-HQ: all HQ CNVs detected (deletion if ratio < 1 and duplication if ratio > 1) affecting targets (CNVs-targets-only.HQ.tsv file)
- cn.mops all CNVs detected (deletion if CN < 2 and duplication if CN > 2)

- CNVkit: all CNVs detected (deletion if CN < 2 and duplication if CN > 2)
- CODEX2: all CNVs detected (deletion if lratio < 2 and duplication if lratio > 2)
- CoNIFER: the tool could not be run on this dataset
- ExomeDepth: all CNVs detected (deletion if Reads-ratio < 1 and duplication if Reads-ratio > 1)
- Control-FREEC: all CNVs detected (deletion if CN < 2 and duplication if CN > 2)
- GATK: all CNVs detected (deletion if CN < 2 and duplication if CN > 2)
- SavvyCNV: all CNVs detected (deletion if relative dosage < 1 and duplication if relative dosage > 1)

All detected CNVs overlapping with the true set and with the correct ploidy (heterozygous or homozygous events) were considered as true positives. Events without any overlaps were considered as false negatives. In a second step, the same analysis was repeated for CNVs overlapping exactly the captured regions found in the validated CNVs. Sensitivity was computed as the number of detected CNVs divided by the total number of validated CNVs; specificity as the number of validated CNVs divided by the total number of detected CNVs.

Parameters used for CNV detection in the retinal disease samples

CNV detection was run separately for each capture kit (1 to 3), as follows:

- OFF-PEAK: default parameters with target BED file, using both, all-targets, and on-targets-only output. Columns “begin-min” and “end-max” were used for additional analysis. For performance analysis, overlapping CNVs detected by on-target and off-target approaches were counted only once. For further investigation with lower

stringency, the following parameters were used: minOntarget=30, maxOntarget=75, minZ=3.

- cn.mops: default parameters
- CNVkit: default parameters with target BED file
- CODEX2: default parameters with target BED file, without chromosome X
- CoNIFER: default parameters with target BED file, with 1 SVD component removed for kit 1 and kit 3. For kit 2, CoNIFER BED file was used with 4 SVD components removed
- ExomeDepth: default parameters
- Control-FREEC: default parameters
- GATK: default parameters with target BED file
- SavvyCNV: default parameters; after running CoverageOffTarget command, SavvyCNV was run with -d 200 for on-target analysis; for off-target analysis, it was run with -d 19800 for kit 1, -d 29800 for kit 2, and -d 27400 for kit 3. For performance analysis, overlapping CNVs detected by on-target and off-target approaches were counted only once.

The computing time for each batch and each tool was recorded. All tools were used on the same machine, a DELL Power Edge R640 with 36 CPUs (Intel Xeon Gold 6150).

SNV filtering for the retinal disease cohort

DNA variants were filtered to be rare, with an allelic frequency lower than 0.01 in gnomAD,[4] as well as in ToMMo,[5] ABraOM,[6] ESP6500,[7] and present in an in-house inventory of sequenced samples. Moreover, they were selected to be of high quality (GQ > 30,

allelic_ratio > 0.25, FS < 25, VQSLOD > -5, and ExcessHet < 50) and to have a predicted impact at the protein level (nonsense, frameshift, missense, canonical splice site or splicing variants [MaxEntScan, SpliceAI and dbscSNV-ADA]). Variants were further selected to affect genes known to cause IRD phenotypes based on OMIM[8] (Table S4).

Detection of potentially pathogenic CNVs in the IRD cohort

The CNVs detected by all tools except controlFREEC (due to too many calls, low specificity) and CNVkit (too many CNVs called for a few specific cases) were first selected to be rare (detected in less than 5 samples per tool) and to affect genes known to cause retinal phenotypes. During the selection process the operator was not blinded with respect to the tool examined, but there was no bias in the selection of calls, which were all considered as equivalent and processed in the same way in the following steps. All genes were first selected from OMIM to have a solid association with IRDs and then filtered to have known mechanism of disease corresponding to loss-of-function (for autosomal recessive and X-linked conditions) or haploinsufficiency (for autosomal dominant phenotypes) (Table S4).

In addition, CNVs linked to autosomal recessive or X-linked phenotypes were retained if:

- they were detected as likely homozygous events (homozygous deletion or duplication defined as ploidy < 0.25 (for deletions), or between 3.25 and 5 (for duplications)
- there were two CNVs detected in the same gene (compound heterozygous)
- there was a rare and deleterious SNV or small indel affecting the same genes (compound heterozygous)

and for autosomal dominant phenotypes if:

- they were detected as likely heterozygous events (ploidy is not 2)

The selected events were then manually filtered to make sure they were: not artefactual (based on IGV visualization of BAM files), affecting exonic regions, and compatible with the phenotype of all affected individuals (Table S3). They were then all validated either through PCR, WGS or MLPA (Table 1). The list of primers used for the PCR validations can be found in Table S11. MLPA analysis for copy number variation detection on *EYS* gene was carried out using MLPA Salsa P328-A2 probemix (MRC Holland, Netherlands). WGS was performed either at CeGaT GmbH (Tübingen, Germany) or at D-BSSE (ETHZ, Basel) on a Novaseq 6000 (CeGaT) or a Novaseq SP flowcell (50-8-8-50, D-BSSE). Libraries were generated with TruSeq DNA PCR-Free kit (Illumina) for CeGaT or with the Watchmaker DNA Library Prep Kit with Fragmentation (Watchmaker Genomics, USA) and indexed with TruSeq™–Compatible Duplex Y Adapters (Integrated DNA Technology) for D-BSSE.

Data processing for the validation on the IRD set

All detected CNVs overlapping with the true set and with the correct ploidy (heterozygous or homozygous events) were considered as true positives. Events without any overlaps were considered as false negatives. In a second step, the same analysis was repeated for CNVs overlapping exactly the captured regions found in the validated CNVs. Sensitivity was computed as the number of detected CNVs divided by the total number of validated CNVs; specificity as the number of validated CNVs divided by the total number of detected CNVs. To avoid double scoring of the same events, the total number of CNVs detected by OFF-PEAK was the sum of non-overlapping CNVs for all targets and of on-target-only analyses. Similarly,

for SavvyCNV, the total number of CNVs was the sum of non-overlapping CNVs from on-target and off-target analyses.

References

1. Geoffroy, V., Herenger, Y., Kress, A., Stoetzel, C., Piton, A., Dollfus, H., and Muller, J. (2018). AnnotSV: an integrated tool for structural variations annotation. Bioinformatics 34, 3572-3574.
2. Robinson, J.T., Thorvaldsdottir, H., Winckler, W., Guttman, M., Lander, E.S., Getz, G., and Mesirov, J.P. (2011). Integrative genomics viewer. Nat Biotechnol 29, 24-26.
3. Mahamdallie, S., Ruark, E., Yost, S., Ramsay, E., Uddin, I., Wylie, H., Elliott, A., Strydom, A., Renwick, A., Seal, S., et al. (2017). The ICR96 exon CNV validation series: a resource for orthogonal assessment of exon CNV calling in NGS data. Wellcome Open Res 2, 35.
4. Karczewski, K.J., Francioli, L.C., Tiao, G., Cummings, B.B., Alfoldi, J., Wang, Q., Collins, R.L., Laricchia, K.M., Ganna, A., Birnbaum, D.P., et al. (2020). The mutational constraint spectrum quantified from variation in 141,456 humans. Nature 581, 434-443.
5. Ogishima, S., Nagaie, S., Mizuno, S., Ishiwata, R., Iida, K., Shimokawa, K., Takai-Igarashi, T., Nakamura, N., Nagase, S., Nakamura, T., et al. (2021). dbTMM: an integrated database of large-scale cohort, genome and clinical data for the Tohoku Medical Megabank Project. Hum Genome Var 8, 44.
6. Naslavsky, M.S., Scliar, M.O., Yamamoto, G.L., Wang, J.Y.T., Zverinova, S., Karp, T., Nunes, K., Ceroni, J.R.M., de Carvalho, D.L., da Silva Simoes, C.E., et al. (2022). Whole-genome sequencing of 1,171 elderly admixed individuals from Sao Paulo, Brazil. Nat Commun 13, 1004.
7. Fu, W., O'Connor, T.D., Jun, G., Kang, H.M., Abecasis, G., Leal, S.M., Gabriel, S., Rieder, M.J., Altshuler, D., Shendure, J., et al. (2013). Analysis of 6,515 exomes reveals the recent origin of most human protein-coding variants. Nature 493, 216-220.

8. Amberger, J.S., Bocchini, C.A., Schiettecatte, F., Scott, A.F., and Hamosh, A. (2015). OMIM.org: Online Mendelian Inheritance in Man (OMIM(R)), an online catalog of human genes and genetic disorders. Nucleic Acids Res 43, D789-798.
