# Supplementary material for: Detection of elusive DNA copy-number variations in hereditary disease and cancer through the use of noncoding and off-target sequencing reads
Source: Am J Hum Genet. 2024 Mar 25;111(4):701–13. doi: 10.1016/j.ajhg.2024.03.001 (PMC11023916; doi:10.1016/j.ajhg.2024.03.001)
Supplement: Document S2. Article plus supplemental information [file mmc13.pdf]

Detection of elusive DNA copy-number variations in hereditary disease and cancer through the use of noncoding and off-target sequencing reads

Authors

Mathieu Quinodoz, Karolina Kaminska, Francesca Cancellieri, ..., Veronika Vaclavik, Hendrik P.N. Scholl, Carlo Rivolta

Correspondence

carlo.rivolta@iob.ch

Copy-number variations are a type of DNA variants that can lead to genetic disease and cancer. Because of important technical limitations, they are usually difficult to identify. We have created a software, OFF-PEAK, which makes use of data that are discarded by conventional procedures to efficiently detect these particular variants.

Quinodoz et al., 2024, The American Journal of Human Genetics *111*, 701–713

April 4, 2024 © 2024 The Author(s).

https://doi.org/10.1016/j.ajhg.2024.03.001

Detection of elusive DNA copy-number variations in hereditary disease and cancer through the use of noncoding and off-target sequencing reads

Mathieu Quinodoz,[1,2,3] Karolina Kaminska,[1,2] Francesca Cancellieri,[1,2] Ji Hoon Han,[1,2] Virginie G. Peter,[1,2,4] Elifnaz Celik,[1,2] Lucas Janeschitz-Kriegl,[1,2] Nils Schärer,[1,2] Daniela Hauenstein,[1,2] Bence György,[1,2] Giacomo Calzetti,[1,2] Vincent Hahaut,[1,2] Sónia Custódio,[5] Ana Cristina Sousa,[5] Yuko Wada,[6] Yusuke Murakami,[7] Almudena Avila Fernández,[8,9] Cristina Rodilla Hernández,[8,9] Pablo Minguez,[8,9] Carmen Ayuso,[8,9] Koji M. Nishiguchi,[10] Cristina Santos,[11,12] Luisa Coutinho Santos,[12] Viet H. Tran,[13,14] Veronika Vaclavik,[13] Hendrik P.N. Scholl,[1,2] and Carlo Rivolta[1,2,3,*]

Summary

Copy-number variants (CNVs) play a substantial role in the molecular pathogenesis of hereditary disease and cancer, as well as in normal human interindividual variation. However, they are still rather difficult to identify in mainstream sequencing projects, especially involving exome sequencing, because they often occur in DNA regions that are not targeted for analysis. To overcome this problem, we developed OFF-PEAK, a user-friendly CNV detection tool that builds on a denoising approach and the use of "off-target" DNA reads, which are usually discarded by sequencing pipelines. We benchmarked OFF-PEAK on data from targeted sequencing of 96 cancer samples, as well as 130 exomes of individuals with inherited retinal disease from three different populations. For both sets of data, OFF-PEAK demonstrated excellent performance (>95% sensitivity and >80% specificity vs. experimental validation) in detecting CNVs from *in silico* data alone, indicating its immediate applicability to molecular diagnosis and genetic research.

Introduction

Targeted next-generation sequencing (NGS) approaches, such as whole-exome sequencing (WES), are widely used to investigate the molecular origin of hereditary disease, cancer, or normal interindividual genetic variability. Small DNA changes such as single-nucleotide variants (SNVs) or short insertions and deletions are often identified as the underlying cause of these disorders or phenotypes (e.g., Töpf et al.,[1] Perea-Romero et al.,[2] Bae et al.[3]). However, in many cases, pathogenic genetic variants consist of DNA rearrangements, such as deletions or duplications, involving dozens to millions of base pairs. These larger events, collectively termed copy-number variants (CNVs), can be responsible for disease in up to 20% of affected individuals, depending on the specific condition and the ethnicity of the cohorts analyzed.[4–6]

CNVs can be detected using specific molecular biology techniques, such as microarray-based comparative genomic hybridization (array-CGH) or multiplex ligation dependent probe amplification (MLPA).[7] However, these analyses involve higher costs with respect to mainstream genomic technologies and are not routinely applied in genetic diagnosis. A few studies have shown that CNVs can be detected by exploiting the information contained in NGS data, such as WES, whole-genome sequencing (WGS), or targeted sequencing (NGS panels). More specifically, they can be inferred using multiple layers of information that are embedded in sequencing data: relative read coverage, split-reads, split pairs, B-allele frequency (BAF) of SNVs, *de novo* assembly, or a combination of these.[8–10]

Approaches based on coverage of captured regions are the most relevant ones when data from WES or from NGS panels are considered, since these experiments are unlikely to include split-reads, split pairs, or enough SNVs for BAF analysis. In these instances, CNVs are detected by comparing the depth of reads aligning to the reference sequence of the human genome, since deletions should in theory result in lower local coverage, whereas duplications should result in increased coverage. However, coverage-based CNV detection faces a major challenge,

[1]Institute of Molecular and Clinical Ophthalmology Basel (IOB), Basel, Switzerland; [2]Department of Ophthalmology, University of Basel, Basel, Switzerland; [3]Department of Genetics and Genome Biology, University of Leicester, Leicester, UK; [4]Department of Ophthalmology, Inselspital, Bern University Hospital, Bern, Switzerland; [5]Department of Medical Genetics, Hospital Santa Maria, Centro Hospitalar Universitário Lisboa Norte (CHULN), Lisbon, Portugal; [6]Yuko Wada Eye Clinic, Sendai, Japan; [7]Department of Ophthalmology, Graduate School of Medical Sciences, Kyushu University, Fukuoka, Japan; [8]Department of Genetics & Genomics, Instituto de Investigación Sanitaria-Fundación Jiménez Díaz University Hospital, Universidad Autónoma de Madrid (IIS-FJD, UAM), Madrid, Spain; [9]Centre for Biomedical Network Research On Rare Diseases (CIBERER), Madrid, Spain; [10]Department of Ophthalmology, Nagoya University Graduate School of Medicine, Nagoya, Japan; [11]NOVA4Health, NOVA Medical School, Faculdade de Ciências Médicas, NMS, FCM, Universidade NOVA de Lisboa, Lisbon, Portugal; [12]Instituto de Oftalmologia Dr Gama Pinto (IOGP), Lisbon, Portugal; [13]Unité d'oculogénétique, Jules Gonin Eye Hospital, University of Lausanne, Lausanne, Switzerland; [14]Centre for Gene Therapy and Regenerative Medicine, King's College London, London, UK

*Correspondence: carlo.rivolta@iob.ch

https://doi.org/10.1016/j.ajhg.2024.03.001.

© 2024 The Author(s). This is an open access article under the CC BY license (http://creativecommons.org/licenses/by/4.0/).

namely the considerable variability in read depth that is normally detected within and across samples. Coverage variability, due to differences in DNA quality, capture efficiency, read mappability, etc., represents in fact an overwhelming source of noise that can easily mask the true signal originating from CNVs.[11] To circumvent this problem, several denoising approaches have already been implemented and incorporated into *in silico* tools, including principal components analysis (PCA) and singular value decomposition (SVD), leading to variable results.[10] These tools are also based on various algorithms and may be adapted to mine specific sequencing sets (e.g., NGS panels, WGS, etc.) or be primarily suitable for particular CNV types (e.g., large CNVs associated with cancer or, conversely, small CNVs associated with Mendelian diseases). Moreover, it can be noted that the degree of user-friendliness and the type of output files differ from tool to tool.[10]

In WES and NGS panels, the template DNA to be sequenced is pre-processed by hybridization capture or by other techniques, in order to select some regions of the genome for downstream analysis while discarding others. Interestingly, however, in addition to sequences from captured regions, the raw output of these experiments also contains many off-target reads, i.e., sequences belonging to portions of the genome that were not selected for further processing but were nonetheless present as contaminants in sequencing libraries. Such sequences may represent in fact up to 60% of the total reads[12] and can be used, in principle, to detect CNVs. We reasoned that this *a priori* unwanted information, in combination with region-specific denoising approaches, could be harnessed to predict the presence of CNVs from NGS data. By exploiting this concept, we have developed OFF-PEAK, a tool that, by taking BAM and BED files as inputs, is capable of performing three essential functions: (1) detecting rare CNVs (in targeted regions, untargeted regions, or both) by using off-target reads, (2) achieving high performance in this task by using only primary data from WES or NGS panels, and (3) providing comprehensive and user-friendly output files. In essence, as described below, we created a robust and versatile CNV detection software that can be used to analyze any generic NGS project.

Material and methods

Samples from human subjects

This study was performed according to the tenets of the Declaration of Helsinki and was approved by the Ethics Committees of the respective Institutions involved: the Ethikkommission Nordwest- und Zentralschweiz, the Commission Cantonale d'Éthique de la Recherche sur l'Être Humain du Canton de Vaud, the Comissão de Ética para a Saúde do Instituto de Oftalmologia Dr. Gama Pinto, the Comité de Ética de la Investigación de la Fundación Jiménez Díaz, the Institutional Review Boards of the Kyushu University Hospital, the Yuko Wada Eye Clinic, and the Nagoya University Hospital. Written informed consent was obtained from all participants or their legal guardians prior to their inclusion in this study. DNA was extracted from the participants' whole-blood or saliva samples.

We selected 130 persons with inherited retinal diseases (IRDs) who did not have a clear molecular diagnosis prior to CNV detection to be used as a validation set for OFF-PEAK. For the quantification of on- vs. off-target reads, we used 22 of these samples, as well as 172 other affected individuals; cross-platform comparison was achieved by analyzing data from 60 additional subjects (51 + 9), as detailed below.

Whole-exome sequencing procedures

WES was performed at CeGaT GmbH. There, sequencing libraries were generated using either the Twist Human Core Exome (kit 1, batch of 11 samples), the Twist Human Core Exome Plus (kit 2, batch of 97 samples), or the Twist Exome 2.0 Panel (kit 3, batch of 22 samples) (Twist Bioscience) following the manufacturers' protocols. Libraries underwent paired-end sequencing on a NovaSeq 6000 (Illumina), resulting in reads of 100 bases. Obtained reads were subsequently processed by our team.

Scoring of on- and off-target reads

We used the CollectHsMetrics command from Picard (v.2.23.8) with default parameters to retrieve the number of mapped reads in targeted regions, near them, or somewhere else. The command was run for the 194 samples (172 + 22) described above, which were also sequenced using the Twist Exome 2.0 panel (kit 3) and Illumina NovaSeq 6000 machines with at least 12 Gb of output per sample. Additionally, the same command was run on 51 samples sequenced using the SureSelect Human All Exon V6 capture kit by Agilent (sequenced on a NovaSeq 6000), as well as 9 samples sequenced using the TruSight One Expanded capture kit by Illumina and sequenced with a NextSeq 500 system (Illumina).

Mapping and variant calling

The raw sequence files were assessed, trimmed, and finally mapped back to the human genome reference sequence (build hg19/GRCh37) using BWA (v.0.7.17). Then, Picard (v.2.14.0-SNAPSHOT) and GATK (v.4.1.4.1) were used to process mapped reads and perform base quality score recalibration and variant calling. DNA variants were processed and scored according to an internal computational pipeline,[13] using ANNOVAR.[14]

Processing of on- and off-target intervals by OFF-PEAK

The 01_targets-offtargets.sh script of OFF-PEAK was used to process the input BED files provided by Twist Bioscience for capture kits 1–3, containing the information relative to targeted regions (–targets option) defined for either the hg19 or the hg38 genome builds (–genome option). The process developed as follows: first, regions smaller than a given threshold (–minOntarget, default 100 bp) were extended to reach this specific threshold, and regions larger than a given value (–maxOntarget, default 300 bp) were split into equal parts to reach a size below this value. Next, off-target regions were defined as the whole reference sequence minus all padded (–paddingOfftarget, default 300 bp) on-target regions. These were then further divided into equal parts if they were larger than a specific value (–maxOfftarget, default 50,000 bp) or discarded if they were smaller than a minimum size (–minOfftarget, default 1 bp). RefSeq exons within both on- and off-target regions were annotated as such in the output BED file. This step required the use of R (annotate-off-targets.R and annotate-targets.R scripts),

without the use of any particular libraries, as well as of the reference genome as a single FASTA file (–ref option).

All parameters were heuristically optimized following the analysis of more than 1,000 internal WES data and can be further optimized for other types of capture kits or sequencing procedures. More specifically, we recommend adapting the maxOfftarget parameter according to the percent of off-target vs. on-target reads, i.e., maxOfftarget = 2 * maxOntarget * (% on-target reads)/(% off-target reads), the value 2 representing a "safety" parameter that takes into account the variability of the coverage for off-target regions. For instance, if the maximum size of on-target regions is 300 bp, we would advise to select a maximum size of off-target regions of 50 kbp for the Twist capture kit, 70 kbp for the Agilent kit, and 60 kbp for the Illumina kit.

Determination of coverage of on- and off-target regions by OFF-PEAK

To compute the number of base coverage to each specific region, OFF-PEAK (02_bam-count.sh script) used the mosdepth software (v.0.3.2),[15] with parameters: –no-per-base, –threads 2, –mapq 50. As input, it used the processed targets (output of 01_targets-offtargets.sh script, –targetsBED), the working directory (–work), the location of the mosdepth software (–mosdepth), and a tab-delimited text file containing one or two columns (BAM file and possibly sample IDs, –listBAM).

CNV detection by OFF-PEAK

For the whole procedure, the following R libraries were used: optparse (v.1.7.3),[16] gplots (v.3.1.3),[17] ExomeDepth (v.1.1.16),[18] pROC (v.1.18.0),[19] and caTools (v.1.18.2).[20] CNV detection was achieved by analyzing each sample separately, according to the following procedure. First, control samples were selected as those displaying high correlation values with respect to the test sample (–mincor, default: 0.9), based on 10,000 randomly selected autosomal target regions passing the requirement on minimum coverage and maximum variance (–minsignal and –maxvar, defaults: 2,500 and −0.2, respectively). The smallest number of control samples selected was 15 (unless there were fewer samples in total), the maximum was 96.

Target regions were then selected to include an annotated exon or to have a minimal size (–minOfftarget, default: 1,000). The signal on such targets was subsequently normalized to their GC content by using the *lm* function from the R Stats package on the signal for the test sample, divided by the average signal from the control samples. This procedure was applied separately for autosomes and for the X chromosome. Normalization of the signal from each target region was also performed with respect to the total coverage from the sample it belonged to (sum of coverages of all regions). This, again, was done separately for autosomes and for the X chromosome. After that, all targets with an average coverage inferior to a pre-defined value or with variance of coverage superior to a threshold were filtered out (–minsignal and –maxvar options).

Noise removal was achieved by using a leave-one-out principal components analysis (LOO-PCA) approach (see supplemental methods for details). Following this denoising process, CNVs were computed on the test sample versus the control samples, excluding control samples with coverage values that were outside of 2 standard deviations from the average of controls. CNVs involving more than one target were detected using the viterbi.hmm function (ExomeDepth package v.0.8.0),[18] with transition probabilities of 0.00005 between a normal copy number state and another state, and of 0.5 to continue in the same state. Single-target events were selected based on the absolute *Z* score (–minZ, default 4), and only if they were not part of events involving multiple targets. Ploidy was determined as corresponding to nearest entire number with the smaller *Z* score with respect to the test sample.

Further details on detection and annotation of CNVs, as well as on the production of output files, are described in the supplemental methods.

Results

Scoring of off-target reads in WES data

We found that a considerable number of contaminant DNA from untargeted regions of the genome (Figure 1A) are present in the sequence files of WES, even when using the latest technologies for DNA capture and parallel sequencing (Figure S1). Specifically, the analysis of 194 WES datasets that were recently generated by our team using a capture kit from Twist (Exome 2.0) revealed that, on average, 47.2% of the reads obtained mapped directly to the targeted regions, 33.6% lay in their close vicinity (within 250 bp), 18.0% mapped to further non-captured regions, and 1.2% did not map anywhere. Since targeted regions and their proximal sequences represent only 5.7% of the human genome, we can expect the coverage of off-target regions to be approximately 80 times lower than that of targeted ones (18.0/(47.2 + 33.6)*0.057 ≈ 1/80). This would imply a coverage of ~2× in off-target regions for a WES having an average coverage of 200×, representing a major source of data and an unexpected opportunity for CNV detection. Following this reasoning, a CNV tool that could identify a CNV affecting a single exon should also, in principle, be able to detect a CNV affecting a ~100 times larger off-target region.

To investigate whether the number and distribution of off-target reads was comparable to those of other capture kits, we analyzed sequencing data produced by using the Agilent (SureSelect) and Illumina (TruSight One) systems. We found indeed very similar values, and specifically that 50.9% and 48.5% of reads mapped to targeted regions, 35.6% and 34.9% to their close vicinities, and 12.7% and 14.2% further in non-captured regions for the Agilent and the Illumina kits, respectively.

Implementation of OFF-PEAK

Based on these data and on the possibility of exploiting off-target reads for CNV detection, we designed a specific workflow, ideally meant to be run on a set of samples sequenced in similar experimental conditions, and condensed it into a single software, OFF-PEAK. The analytical process of this tool was divided into four main steps; (1) target region pre-processing, (2) counting reads and coverage, (3) CNV detection, and (4) CNV annotation and graphical representation (Figure 2, details in supplemental methods).

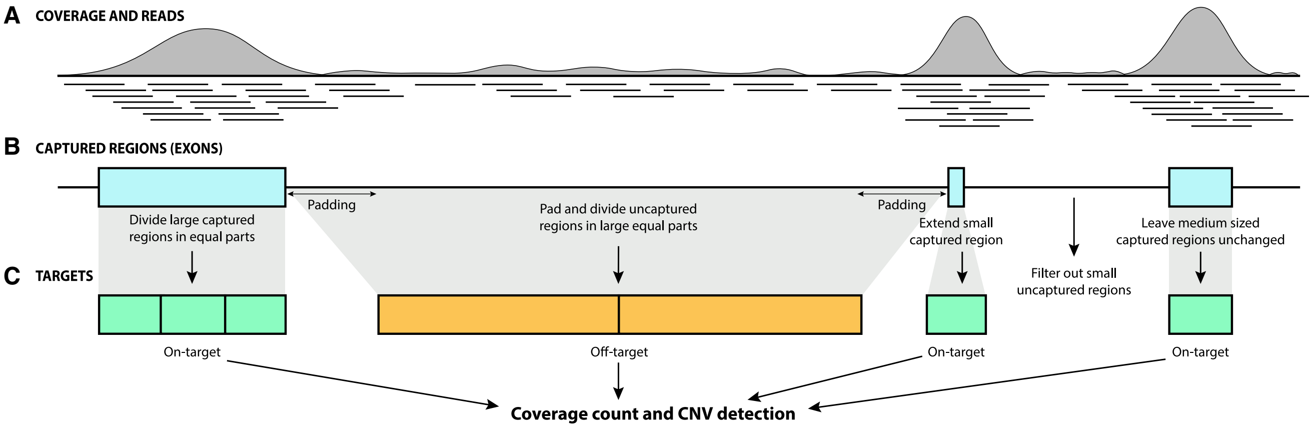

Figure 1. Use of uncaptured reads contaminating WES or NGS panels' libraries for the detection of CNVs
(A) Schematic view of a typical coverage of a genomic region, following targeted NGS. Individual reads are represented by thin horizontal lines and coverage by grey-shaded areas. Since, at the pre-sequencing stage, capturing probes target only specific sequences of the genome (e.g., exons, in blue, in B), coverage tends to be higher for such regions of interest. However, target selection and downstream fragment purification are not 100% efficient, and DNA fragments that lie in uncaptured regions are frequently co-purified along with fragments from targeted areas.
(B and C) Regions of the genome that are targeted by a capturing system, along with their size and position, can be used to define on-target (green boxes) and off-target (orange boxes) sets for further computing the presence of CNVs occurring in captured areas, in uncaptured areas, or in both. Other procedures to improve CNV calling, such as intron padding and resizing of targets, are also shown.

As part of the first step, in order to harvest as much information as possible from off-target reads, we had OFF-PEAK divide the genome based on captured and uncaptured regions (Figure 1B). This was achieved by processing the file containing the coordinates of the targeted sequences that are usually provided by the vendor of the capture kit (BED file). Then, both uncaptured and captured regions were subdivided in parts of similar lengths, defining "on-target regions" and "off-target regions," respectively (Figure 1C). Sizes of these latter regions were purposely set to be larger than those from the former regions, to compensate for the higher coverage displayed by exonic/captured stretches of DNA and allow for a similar number of reads to be present within any given region. In other words, we performed differential partitioning to increase the sensitivity to differences in coverage for every single DNA stretch, and specifically to allow the detection of partial exonic rearrangements. In addition, within off-target sets, we excluded DNA regions that were in close vicinity to on-target regions (default range: 300 bp from each side). Again, the goal of this padding process was to allow for a better identification of differences in coverage in off-target regions, since the high number of reads in these proximal regions, a byproduct of exonic capture, would hide the true coverage value of introns (Figure 1B). Then, small captured regions were extended *in silico* to result in larger on-target regions, using these byproduct reads to allow reliable estimation of coverage (Figures 1B and 1C). Finally, all regions were annotated with exons from RefSeq transcripts for further use.

As a second step, we used the mosdepth software[15] on BAM files to determine the coverage of each on-target and off-target region, for each sample. We then merged these individual data to produce a single coverage file, which contained standardized coverage information relative to all regions.

Step 3 involved the actual detection of CNVs, based on the comparison between every single sample with samples from the rest of the pool (used as controls). Specifically, we first selected all controls that showed highly correlated coverage with the test sample (based on 10,000 randomly selected regions, see material and methods) and then normalized all coverage values based on each sample's total coverage and each region's GC content, using a regression method (details in material and methods). We subsequently filtered the on- and off-target regions based on average coverage and standard deviation to remove outlier regions with insufficient signal or with very high variation in coverage, mostly representing regions with high homology or repeats such as telomeres, centromeres, and pseudogenes. After that, for the test sample we operated a random selection of 1,000 on-target or off-target regions. To half of them, we assigned an artificial coverage corresponding to 50% of the real one, simulating a heterozygous deletion, and to the remaining 500 a 150% coverage, to simulate a heterozygous duplication. OFF-PEAK then applied the leave-one-out PCA (LOO-PCA) method associated with these artificial CNVs to compute how many principal components (PCs) need to be removed to achieve the best performance in retrieving all 1,000 artificial CNVs (see supplemental methods for details on this selection). Noise removal using LOO-PCA is very similar to PCA-based methods, the difference being that the test sample is not included in the computation of PCs and is simply projected on them afterward. By this method, the variation in coverage resulting from the presence of a true CNV in the test sample is not used to build the PCs and therefore the signal is not lost when PCs are removed for noise

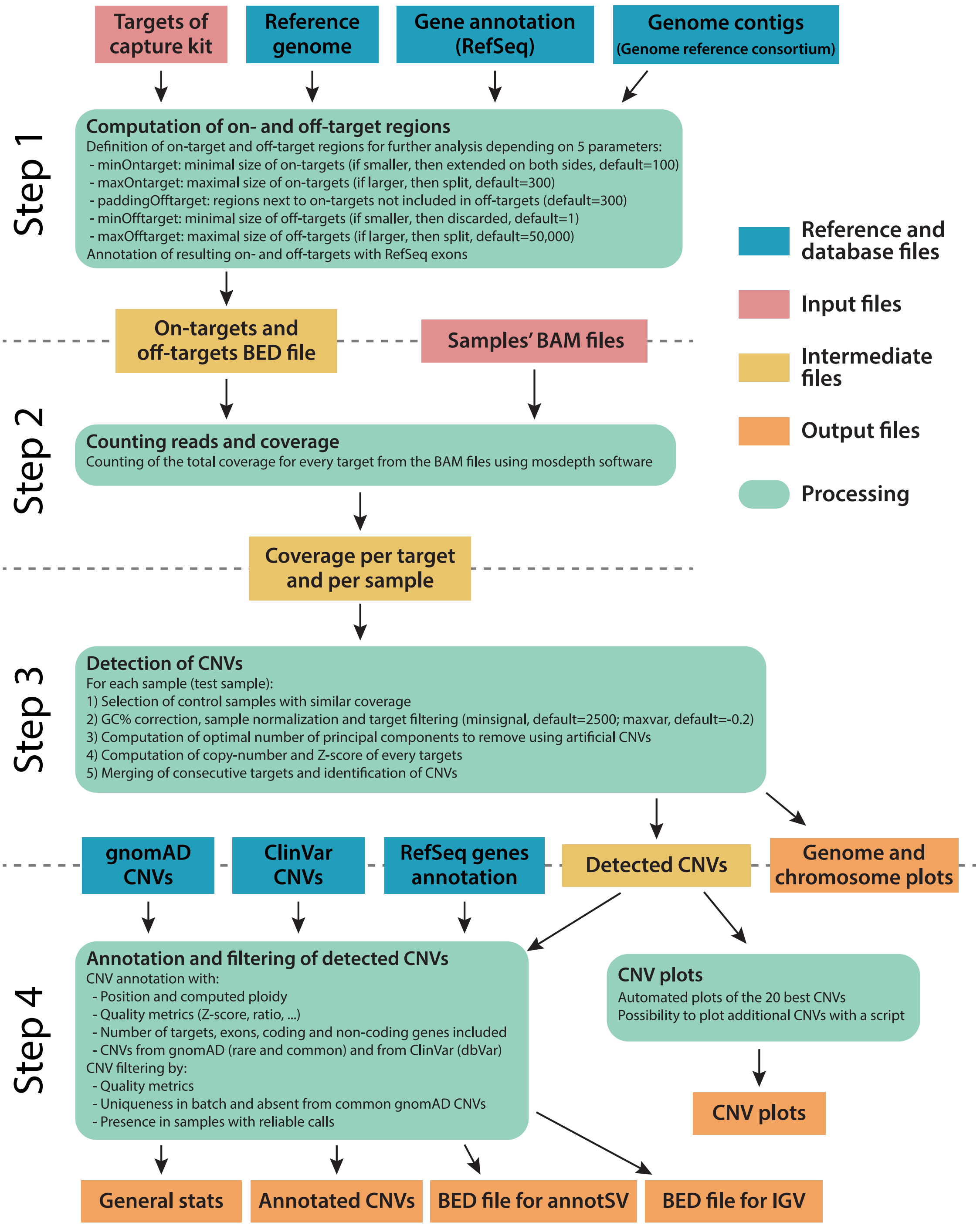

Figure 2. Outline of the procedures used by OFF-PEAK
The workflow is divided into four main steps: (1) processing of target regions, (2) counting reads and coverage, (3) CNV detection, and (4) CNV annotation and graphical representation.

correction. Our analysis showed that computing PCs only on control samples (not including the test sample) results in higher robustness in CNV detection. In other words, CNV signals remain strong even following the removal of multiple PCs and this is, in fact, a signature feature of OFF-PEAK. As an example to illustrate the difference between PCA and LOO-PCA, we selected a heterozygous deletion affecting 20 exons that was detected in an individual sequenced with the capture kit 1. When no PC is removed, the CNV is detectable by visual inspection but cannot be scored as a true CNV since it lies within the range of experimental uncertainty, represented by the gray area in Figure 3A. When an increasing number of PCs is removed with standard PCA, the noise decreases, but so does the signal originating from the true CNV (green squares and blue circles), to the point that it is, in the end, completely

lost. Conversely, with LOO-PCA, the noise progressively decreases with an increasing number of PCs removed, but the signal originating from the CNV does not. To better score this phenomenon, we assessed the detection performance on artificial CNVs when PCs are removed with PCA or LOO-PCA. We found that performance of LOO-PCA remains robust, even when many PCs are removed, whereas it drops quickly with PCA. This effect was found to be similar for samples sequenced within small (Figure 3B) and large (Figure 3C) batches. After noise removal using PCs, CNVs were detected by comparing PC-processed coverage values of the test sample vs. control samples. CNVs affecting multiple consecutive targets were further inferred with a Hidden Markov Model (HMM) approach using the Viterbi algorithm,[18] as described in the material and methods.

As a fourth step, multiple output files were created: text files annotating all detected CNVs with various features, files compatible with AnnotSV[21] and the Integrative Genomics Viewer (IGV),[22] graphical representations of CNVs, as well as genome-wide and chromosome-specific coverage plots (Figure S2). These files allow the user to analyze all identified CNVs in detail and, if needed, take advantage of additional software for further investigations. The graphical representation of the detected CNVs (Figure S3), in particular, allows for a quick visualization of the CNV and its neighboring reads (including off-target reads, frequent [benign] CNVs from the gnomAD database, and pathogenic CNVs from the ClinVar database). It also allows a better representation of the noise level around a CNV, compared to standard quality metrics.

Moreover, we implemented an additional filter, called OFF-PEAK-HQ, to select "high-quality" CNVs, i.e., structural variants that are called with higher confidence and are more likely to represent true positive events (see supplemental methods for details).

Validation of the tool on the ICR96 dataset

As a first testing ground for OFF-PEAK, we selected the ICR96 reference dataset (ICR96 exon CNV validation series), comprising a collection of 96 cancer samples for which 26 genes were sequenced by NGS. All their exons were further experimentally investigated for the presence of copy-number events using MLPA.[23] This set is routinely used for estimating the performance of exon-CNV calling software on NGS data. As a comparison set, we selected eight widely used tools that were developed for CNV detection starting from WES or panel-NGS reads,[10] and specifically: cn.mops,[24] CNVkit,[25] CODEX2,[26] CoNIFER,[27] Control-FREEC,[28] ExomeDepth,[18] GATK gCNV,[29] and SavvyCNV.[30] All these tools were run by using their default parameters, as recommended by their respective developers.

We started by scoring the number of CNVs detected by each tool that overlapped with the true CNVs found by MLPA for at least one captured region, considering the correct predicted ploidy as well. At the end of the process, OFF-PEAK was the only tool that retrieved all of the 68 CNVs validated by MLPA (sensitivity = 100%), with a specificity of 56.7% (Figure 4A; Tables S1 and S2). Three other tools, SavvyCNV, ExomeDepth, and GATK gCNV, displayed very good sensitivity as well (>92%), missing only 2, 4, and 5 CNVs, respectively, but had relatively low specificity (9.8%, 29.6%, and 1.4%, respectively) (Figure 4A; Table S2). The remaining five tools all displayed much lower sensitivity (<36%), clearly separating from the previous ones, while one did not produce any output, because the number of target regions per chromosome was lower than the minimal required input (Figure 4A; Table S2).

We then repeated the analysis by scoring only predicted CNVs that overlapped fully with the captured regions of true CNVs. Sensitivity was slightly lower for all well-performing tools (OFF-PEAK [−4.4%], ExomeDepth [−4.4%], GATK gCNV [−5.8%], and SavvyCNV [−4.4%]), usually because they detected the correct event but missed one captured region or included one adjacent extra region with normal ploidy (Figure S4; Table S2).

When the OFF-PEAK-HQ filter was deployed, specificity increased from 56.7% to 86.8%, at the price of a slightly lower sensitivity, which decreased to 97.1%, due to two CNVs that did not have sufficient quality to be called with high confidence (Figure 4A; Table S2).

Validation on WES from individuals with rare diseases

As an additional test for evaluating the performance of our tool, we gathered WES data from 130 individuals with inherited retinal diseases (IRDs), for whom no causative SNVs or small insertions or deletions (indels) had been found (Table S3). IRDs are rare Mendelian disorders for which mutations in any one of multiple disease-associated genes are at the same time a sufficient and necessary cause of the condition.[31] Specifically, every person with IRD necessarily bears in their genome one (dominant, mitochondrial, X-linked in males) or two (recessive) pathogenic variants, and failure to detect them is generally attributed to technical limitations. The samples analyzed were from individuals of various ethnicities, recruited in Switzerland (n = 79), Portugal (n = 29), and Japan (n = 22). Since, unlike the ICR96 set, true CNVs were not known, we performed a CNV discovery phase within the 132 genes associated with IRDs (Table S4). Using all tools considered above, 1,743 CNVs were identified (Table S5), which were further filtered to be compatible with each gene's inheritance mode (see supplemental methods). CNVs detected by controlFREEC (869 events on 130 exomes) and by CNVkit (75 events, of which 72 in 3 out of 130 exomes) were not considered for the next step, since they represented either falsely positive or artifactual data (see supplemental methods and Table S5). The remaining 556 CNVs were manually examined, looking at the number and the distribution of mapped reads, and then curated based on the correlation between genotype (gene affected by a given CNV) and phenotype (specific clinical signs possibly resulting from the inactivation of that gene). The final list

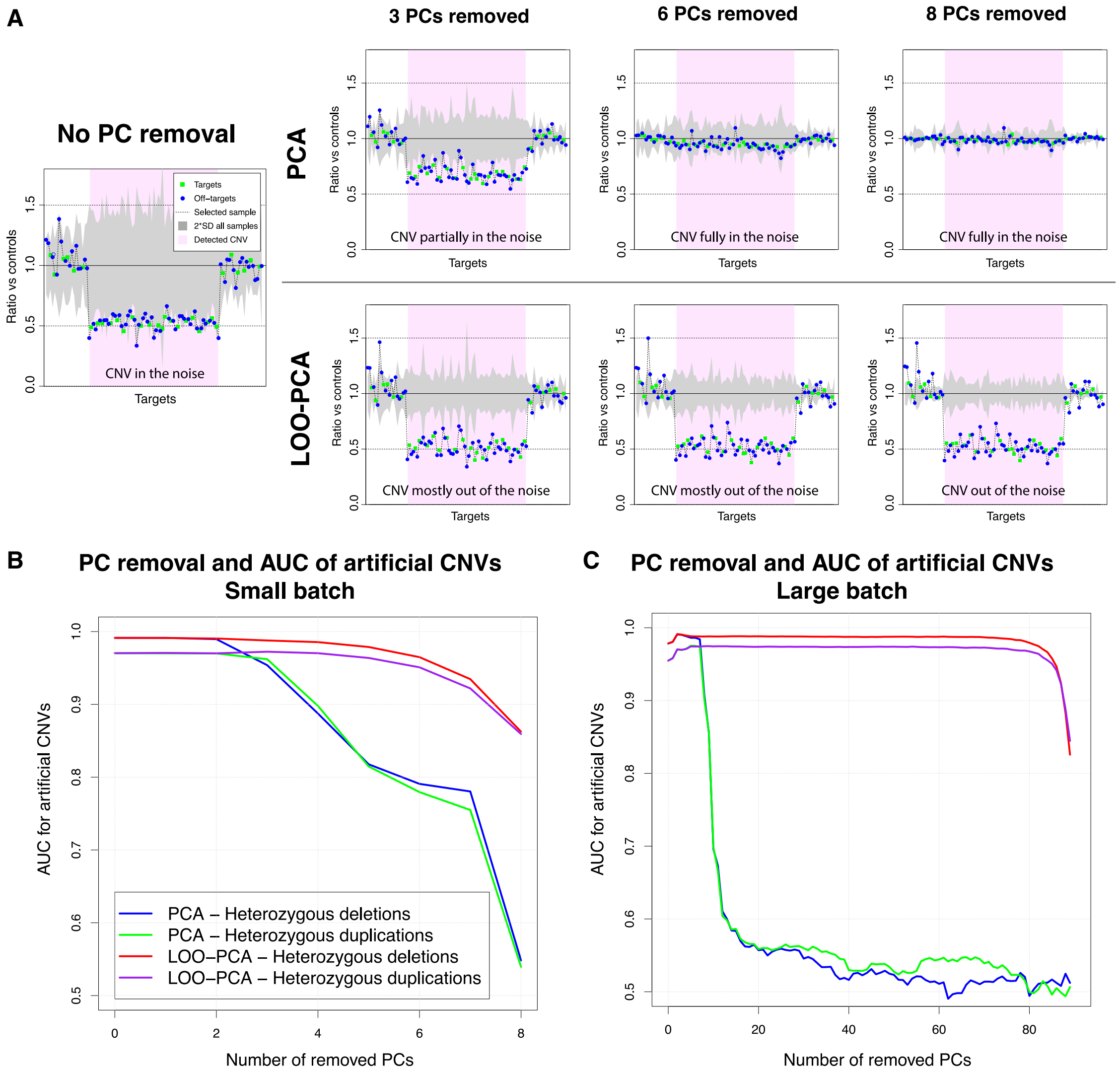

Figure 3. Denoising approaches for CNV detection

(A) Example of the effect of principal components (PC) removal on the detection of a CNV, using PCA or LOO-PCA. Plots indicate coverage for each target along a common DNA stretch. When no PC is removed, coverage (dotted line) of the CNV (in this case, a heterozygous deletion) falls into the normal range of variation of 10 control samples (gray area) and cannot be automatically detected. Removal of initial PC components reduces the noise linked to normal variation of coverage; however, when standard PCs removal is applied, it also reduces the amplitude of the true signal linked to the real CNV (top row). This is not the case for PC removal using the LOO-PCA approach, which reduces the noise associated with normal variation of coverage but does not affect the true signal (bottom row).

(B) OFF-PEAK's performance in retrieving artificial CNVs (500 heterozygous deletions and 500 heterozygous duplications) by removing an increasing number of PCs (x axis) using either PCA or LOO-PCA for a small batch of 11 samples. Performance values are presented as area under the curve (AUC) of a receiver operating characteristic (ROC) curve.

(C) Same procedure depicted in (B), but for a larger WES batch (97 samples).

of candidates included 37 CNVs in 34 individuals, which were all experimentally validated and found to correspond to real events by polymerase chain reaction (PCR), WGS, MLPA, or a combination of these techniques (Table S6).

We therefore considered these 37 CNVs as true positives and computed the performance of OFF-PEAK vs. that of the other tools on these specific data, first by taking into account CNV calls having the correct ploidy and overlapping with true CNVs. OFF-PEAK outputted a total of 138 predictions, including the 37 true positive ones, corresponding to a sensitivity value of 100% and a specificity of 27.4% (Figure 4B; Tables S2 and S7). OFF-PEAK-HQ detected 35 events out of 37 as high-quality CNVs, resulting in a sensitivity of 94.6% and a specificity of 79.5% (Figure 4B). Interestingly, the two missed CNVs belonged to the two samples with the lowest maximum pairwise correlations, which emphasizes the importance of using high-quality DNA as starting material and the need for a sufficiently large batch of samples in order to obtain a reliable analysis. As for the ICR96 data, ExomeDepth, GATK gCNV, and SavvyCNV also displayed good performances, although inferior to OFF-PEAK, missing 7, 7, and 13

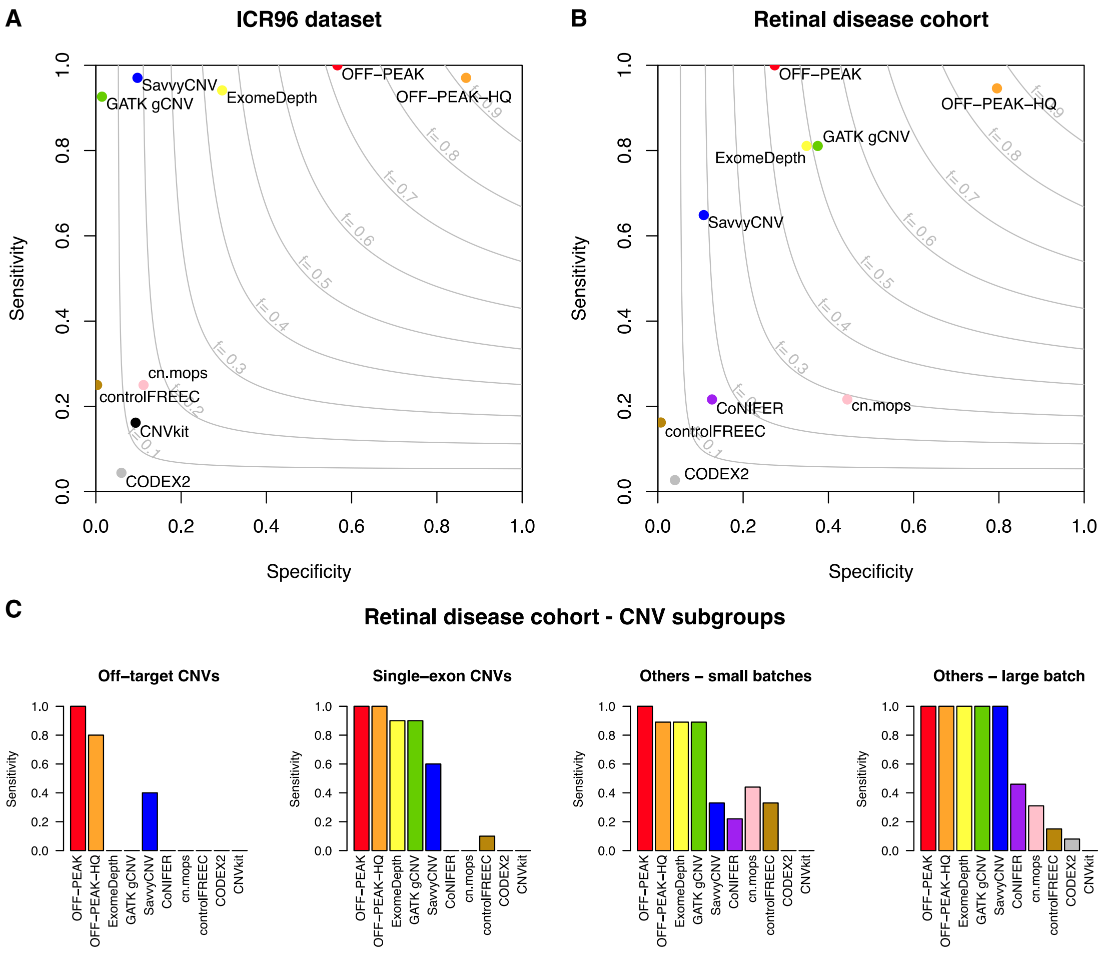

Figure 4. Performance of OFF-PEAK and other tools with respect to different testing sets
(A) Specificity-sensitivity plot for the ICR96 dataset of 96 cancer samples, on 68 validated CNVs.
(B) Specificity-sensitivity plot for the cohort of 130 individuals with retinal phenotypes, on 37 validated CNVs. The curves in light gray represent the F-score or harmonic mean of sensitivity and specificity.
(C) Bar plots of sensitivities for the latter cohort, stratified according to the type of CNVs considered: off-target (e.g., noncoding), on-target single-exon, small batches (kits 1 and 3), and large batch (kit 2, see supplemental methods).

CNVs, respectively (sensitivity = 81.1%, 81.1%, and 64.9%; specificity = 34.9%, 37.5%, and 10.8%, respectively) (Figure 4B). Of note, for all tools considered, specificity values were potentially underestimated in this analysis, since it is likely that other true CNVs, apart from the validated ones, were detected *in silico* but were not considered as true positives, since they were not judged to be causative of the disease.

As before, we repeated the analysis by considering only predicted CNVs that strictly overlapped with all captured regions of the true events. OFF-PEAK and SavvyCNV maintained the same performance, with all CNVs completely overlapping with the true ones. ExomeDepth failed to detect complete overlap for one CNV, although it detected it with partial overlap (−2.7% in sensitivity), and GATK gCNV did the same for 4 CNVs (−10.8% in sensitivity) (Figure S4; Table S2).

The graphical output of OFF-PEAK for validated CNVs is shown in Figure S3, and selected examples can be seen in Figure 5.

Detailed performance results

Heterozygous CNVs are notoriously difficult to detect, especially with respect to their homozygous counterparts, due to a lower difference in coverage compared to controls. OFF-PEAK identified all 25 heterozygous CNVs from the retinal disease cohort with high performance (vs. 21 for ExomeDepth and GATK gCNV, the second-best performers; Table S8, example in Figure 5A).

Similarly, single-exon events are generally more difficult to identify because of a lower overall read coverage. This is also the case for CNVs affecting only parts of an exon, since differences in coverage involve only a subset of a captured region. The ICR96 set comprised 25 single-exon CNVs, and OFF-PEAK was able to detect them all (sensitivity = 100%, Table S2). Three other tools—SavvyCNV, ExomeDepth, and GATK gCNV—had good performances, detecting 24, 22, and 21 of them (96%, 88%, and 84% sensitivity, respectively) (Table S2). The other tools tested displayed only 20% sensitivity or lower. In the retinal disease cohort, there were 10 single-exon events, including one partial exonic deletion.

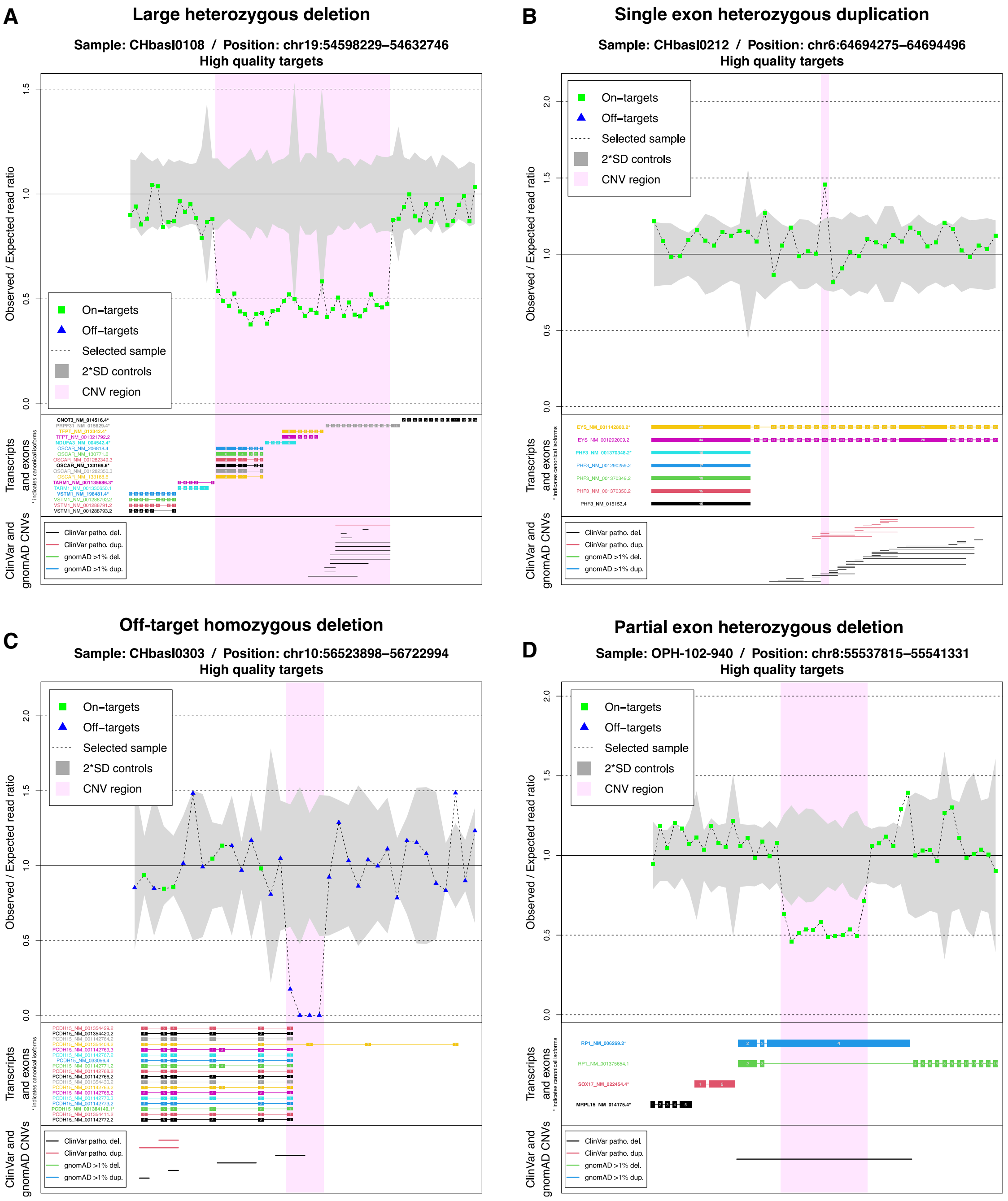

Figure 5. Four relevant examples of OFF-PEAK graphical outputs

(A) Heterozygous deletion affecting 13 exons of *PRPF31* and 3 other genes for CHbasl0108 with retinitis pigmentosa.
(B) Heterozygous duplication affecting exon 35 of *EYS* for CHbasl0212 with retinitis pigmentosa.
(C) Homozygous deletion affecting a non-coding and non-covered exon of *PCDH15* for CHbasl0303 with Usher syndrome type I.
(D) Heterozygous deletion affecting only a part of exon 4 of *RP1* for OPH-102-940 with retinitis pigmentosa.

Again, OFF-PEAK could identify all of them (vs. 9 identified by ExomeDepth and GATK gCNV and 6 by SavvyCNV; Figure 4C; Table S8, example Figures 5B–5D).

Another type of challenging CNVs are those occurring in off-target regions, since they have much lower coverage with respect to targeted ones. In the IRD set, OFF-PEAK detected all of the 5 off-target CNVs (vs. 2 identified by SavvyCNV; Figure 4C; Table S8, example in Figure 5C). These CNVs involved non-coding exons comprising the 5′ UTR of *EYS*, *PRPF31*, and *PCDH15*. Overall, ExomeDepth and GATK gCNV missed all CNVs affecting non-coding exons that were not targeted for DNA capture. This was expected, given that ExomeDepth analyzes only coding exons and GATK gCNV solely uses targeted regions as reference (Figure 4C; Table S8). SavvyCNV detected some CNVs in non-captured regions but missed CNVs in samples from

smaller batches (Figure 4C; Table S3, see supplemental methods for details on batches). This is consistent with previous analyses showing that SavvyCNV needs more than 50 samples to achieve high performances.[30]

We also assessed the presence of split reads by manually inspecting the regions containing validated CNVs using the IGV software.[22] In 73% of all cases (n = 27), no split read could be found, and precise breakpoints could not be resolved (Table S8). In 13.5% of cases (n = 5), only one split read was identified and, finally, in an additional 13.5% of cases more than 1 (precisely, 20 or more) split reads were found, involving the same breakpoint, which was present in a captured region (Table S8).

Finally, we evaluated all tools in terms of time needed to compute the three WES batches used in the IRD tests. For the largest batch (97 samples), computing time ranged from 4.8 to 34.3 h. Among the tools with good performance, GATK gCNV was the fastest (6.8 h), while OFF-PEAK took 21.2 h to complete the analysis. For the smaller batches (11 and 22 samples), the time needed was of 5 h or less for most tools (Figure S5).

Clinical classification of the CNVs detected and further analyses of IRD data

When evaluated for their pathogenicity, 34 out of the 37 validated CNVs detected by OFF-PEAK in the IRD set were classified as likely pathogenic or pathogenic according to the American College of Medical Genetics and Genomics (ACMG) guidelines[32,33] (Table S6). The most frequently affected genes were *EYS* (n = 11), *PRPF31* (n = 5), and *USH2A* (n = 3). Thirteen CNVs were found in genes for which rare and deleterious small variants had already been detected, presumably *in trans* (Table S9). A homozygous deletion of exon 9 of *DRAM2* (GenBank: NM_001349884.2), resulting in an in-frame deletion of 30 amino acid residues, was classified to be of uncertain significance due to insufficient evidence for pathogenicity, while a duplication affecting exons 1 to 9 of *RCBTB1*, occurring in two affected individuals, was classified as likely benign, because it still allows for a complete copy of the gene to be present.

To further investigate all genomes for which causative mutations had not yet been identified and to indirectly test the adaptability of our tool to particular datasets/conditions, we performed a supplementary OFF-PEAK analysis with lower stringency (see supplemental methods). This led to the detection of two additional likely pathogenic CNVs: a partial heterozygous deletion of exon 6 of *ABCA4* in LL359, who also harbors a likely pathogenic missense variant in the same gene (GenBank: NM_000350.3; c.1749G>C [p.Lys583Asn]), and an apparent partial heterozygous deletion of exon 4 of *RP1* in YWC267, which was in fact the sign of a homozygous insertion of an *Alu* element—a frequent pathogenic variant found in the Japanese population (Table S3).[34] Both events were validated by PCR and Sanger sequencing. Of note, these events were also identified by the Scramble software,[35] which detects mobile element insertions (MEIs) and small deletions by identifying clusters of soft-clipped reads.

Discussion

Short-read NGS procedures, including targeted and whole-exome sequencing, are the most commonly used techniques in molecular medical genetics, in particular to detect germline DNA variants that are associated with rare hereditary conditions or somatic mutations leading to cancer. However, despite being tremendously effective in identifying single-nucleotide variants or small pathogenic events, NGS panels and WES perform rather poorly in detecting CNVs. This is not due to a lack of primary information contained in sequencing data but, rather, to the fact that such information is not routinely exploited during data analysis. Specifically, most algorithms focus on coverage of captured regions and use this value as a proxy of ploidy, therefore discarding the data associated with the mapping of off-target reads.

Conversely, OFF-PEAK makes primary use of this type of contaminating data. It has been previously shown that off-target reads can be considered for the detection of CNVs in off-target regions, and a few tools, such as CNVkit,[25] SavvyCNV,[30] cnvOffSeq,[36] and CopywriteR,[37] already make use of them for this purpose. The main differences between OFF-PEAK and existing algorithms consist of two specific and important points. The first is the use of LOO-PCA in order to solve the most critical confounding factor in CNV detection: coverage variability. This noise is intrinsic to any NGS panel or WES, and therefore cannot be completely eliminated at the experimental level. LOO-PCA allows for a high reduction of such noise while keeping most of the signal related to variations in coverage that are linked to the presence of CNVs. In more technical terms, this denoising approach excludes the sample that is under investigation from the calculation of principal components of the coverage data (representing the actual noise), allowing real signal from CNVs not to be lost when PCs are removed by the denoising process. Specifically, the variance associated with coverage values for targets included in a CNV is smaller if the test sample is not taken into consideration. Therefore, such targets have a smaller correction associated with the removal of the first PCs, compared to standard PCA. In principle, it would be advisable not to include multiple members of the same family in the same batch, since such members would be used as controls in the LOO-PCA denoising procedure, and rare CNVs shared with the test sample could result in decreased true signal. In practice, however, it seems that OFF-PEAK's denoising procedure is rather insensitive to this effect, as our test on the IRD large batch showed for instance that up to 7% of samples from the same batch could carry the same rare heterozygous deletion in the gene *DMBT1* without interfering with the call of the CNV itself (Table S10).

The second advantage of OFF-PEAK with respect to other software is the pre-processing of captured and uncaptured regions, which allows for a better scoring of CNVs by the

use of both on- and off-target reads. For targeted regions, DNA sequences are subdivided into bins of similar sizes, regardless of the total length of a consecutive captured stretch, defining on-target intervals. This operation normalizes the signal from such regions (usually exonic sequences) and allows the identification of both small and large CNVs. It also allows the recognition of events that affect captured sequences only in part (e.g., intra-exonic CNVs, Figure 5), which are usually difficult to detect. With respect to non-targeted regions, the same process is applied, although the size of the bins is set to be larger. Such a procedure allows harvesting enough reads for reliable CNV calls and, at the same time, to normalize them with respect to data from captured regions. Most importantly, OFF-PEAK operates a "padding" procedure on off-target regions that are immediately proximal to captured sequences. This process prevents the true signal from off-target regions to be masked (overscored) by the high number of reads covering such non-exonic flanking sequences, which are present in NGS data by virtue of their partial matches with capturing probes at pre-NGS stages. Moreover, unlike other tools, our software is particularly effective at identifying CNVs involving isolated small exons (or captured regions). This is possible because of an OFF-PEAK-specific process, which artificially extends the actual captured sequence for such small regions on both their 5′ and 3′ ends and provides increased sensitivity in coverage detection. All these processes, based on the analysis of off-target regions, also permit restricting the number and size of candidate regions harboring CNV-induced breakpoints and facilitate their identification by molecular biology techniques (e.g., by PCR), even in the absence of split-reads.

When tested on data from 96 cancer samples, OFF-PEAK had the highest performance, detecting all of the 68 MLPA-validated CNVs (100% sensitivity). Since CNVs in this dataset involve on-target regions, the advantage of OFF-PEAK over other tools was mainly due to the use of LOO-PCA, rather than the scoring of off-target reads. Some tools, such as ExomeDepth, SavvyCNV, and GATK gCNV, showed high sensitivity as well, although lower than that displayed by OFF-PEAK. In addition, OFF-PEAK also achieved the highest specificity of all tools considered.

Similarly, when tested on WES data from 130 individuals with hereditary retinal diseases, OFF-PEAK was the only tool that could identify all 37 experimentally validated CNVs affecting genes linked to such conditions. In this case, however, such a high performance could be attributed to the specific use of the information contained in off-target reads. This is evidenced, for instance, by the fact that most CNVs detected solely by our tool were located in untargeted regions. In terms of specificity, OFF-PEAK had a similar performance with respect to the other software, identifying likely causative CNVs in 32 out of 130 affected individuals (24.6%). Conversely, OFF-PEAK-HQ displayed the highest specificity (79.5%), with only a limited reduction in sensitivity with respect to OFF-PEAK (−5.4%). This high specificity is likely due to a more stringent filtering of CNVs, based on various metrics, since this is the only difference between OFF-PEAK and OFF-PEAK-HQ.

Like all the tools evaluated in this study, OFF-PEAK does not use information deriving from split reads to refine breakpoints of CNVs, which is limiting its capacity to identify precise chromosomal junctions. However, because of the paucity of split reads that are normally present in targeted sequencing data, we decided not to use such information, also considering that the presence of split reads can be individually assessed by using dedicated software (e.g., IGV)[22] on the chromosomal regions identified by OFF-PEAK. In addition, even by using coverage information only, our tool demonstrated elevated performances in detecting true CNVs.

In summary, our tests showed that specific strong points of OFF-PEAK are related to the identification of types of events that are difficult to detect by other *in silico* methods, such as heterozygous CNVs, single-exon CNVs, intraexonic events, and CNVs occurring in non-targeted regions of the genome.

CNV rearrangements currently represent one of the most common yet elusive types of pathogenic genotypes in medical and cancer genetics, especially when mainstream sequencing procedures such as WES and targeted NGS are used. By using a specific denoising algorithm, a tailored scoring of different genomic regions and, most importantly, by exploiting the information contained in off-target NGS reads, we created a software that can analyze data from such experiments to detect the presence of small to very large rearrangements with high performance. Our hope is that OFF-PEAK will contribute to a more robust and sensitive detection of pathogenic CNVs, helping molecular diagnosis and basic genetic research alike.

Data and code availability

The OFF-PEAK code is available at https://github.com/mquinodo/OFF-PEAK. The code used for the development of OFF-PEAK and the testing of other tools is available at https://github.com/mquinodo/OFF-PEAK-publication.

Supplemental information

Supplemental information can be found online at https://doi.org/10.1016/j.ajhg.2024.03.001.

Acknowledgments

This work was supported by the Swiss National Science Foundation (grant #176097 to C.R.) and by the Swiss RetinAward (to M.Q.). The authors would like to thank the people with IRDs and their families for their participation in this study. The authors are also grateful to Prof. Nazneen Rahman at The Institute of Cancer Research in London for providing access to the ICR96 data, to Mendurim Rashiti and Marc Perea for IT support, to Beryl Macé for

suggestions, and to Sitta Föhr for her careful revision of this manuscript.

Declaration of interests

The authors declare that there is no conflict of interest.

Received: October 23, 2023
Accepted: March 1, 2024
Published: March 25, 2024

Web resources

ClinVar, https://www.ncbi.nlm.nih.gov/clinvar
GenBank, https://www.ncbi.nlm.nih.gov/genbank/
gnomAD, https://gnomad.broadinstitute.org
UCSC, http://genome.ucsc.edu/cgi-bin/hgTables

References

1. Töpf, A., Johnson, K., Bates, A., Phillips, L., Chao, K.R., England, E.M., Laricchia, K.M., Mullen, T., Valkanas, E., Xu, L., et al. (2020). Sequential targeted exome sequencing of 1001 patients affected by unexplained limb-girdle weakness. Genet. Med. *22*, 1478–1488.
2. Perea-Romero, I., Gordo, G., Iancu, I.F., Del Pozo-Valero, M., Almoguera, B., Blanco-Kelly, F., Carreño, E., Jimenez-Rolando, B., Lopez-Rodriguez, R., Lorda-Sanchez, I., et al. (2021). Genetic landscape of 6089 inherited retinal dystrophies affected cases in Spain and their therapeutic and extended epidemiological implications. Sci. Rep. *11*, 1526.
3. Bae, J.S., Kim, N.K.D., Lee, C., Kim, S.C., Lee, H.R., Song, H.R., Park, K.B., Kim, H.W., Lee, S.H., Kim, H.Y., et al. (2016). Comprehensive genetic exploration of skeletal dysplasia using targeted exome sequencing. Genet. Med. *18*, 563–569.
4. Retterer, K., Scuffins, J., Schmidt, D., Lewis, R., Pineda-Alvarez, D., Stafford, A., Schmidt, L., Warren, S., Gibellini, F., Kondakova, A., et al. (2015). Assessing copy number from exome sequencing and exome array CGH based on CNV spectrum in a large clinical cohort. Genet. Med. *17*, 623–629.
5. Gambin, T., Akdemir, Z.C., Yuan, B., Gu, S., Chiang, T., Carvalho, C.M.B., Shaw, C., Jhangiani, S., Boone, P.M., Eldomery, M.K., et al. (2017). Homozygous and hemizygous CNV detection from exome sequencing data in a Mendelian disease cohort. Nucleic Acids Res. *45*, 1633–1648.
6. Pfundt, R., Del Rosario, M., Vissers, L.E.L.M., Kwint, M.P., Janssen, I.M., de Leeuw, N., Yntema, H.G., Nelen, M.R., Lugtenberg, D., Kamsteeg, E.J., et al. (2017). Detection of clinically relevant copy-number variants by exome sequencing in a large cohort of genetic disorders. Genet. Med. *19*, 667–675.
7. Hills, A., Ahn, J.W., Donaghue, C., Thomas, H., Mann, K., and Ogilvie, C.M. (2010). MLPA for confirmation of array CGH results and determination of inheritance. Mol. Cytogenet. *3*, 19.
8. Zhao, M., Wang, Q., Wang, Q., Jia, P., and Zhao, Z. (2013). Computational tools for copy number variation (CNV) detection using next-generation sequencing data: features and perspectives. BMC Bioinf. *14* (*Suppl 11*), S1.
9. Zare, F., Dow, M., Monteleone, N., Hosny, A., and Nabavi, S. (2017). An evaluation of copy number variation detection tools for cancer using whole exome sequencing data. BMC Bioinf. *18*, 286.
10. Gabrielaite, M., Torp, M.H., Rasmussen, M.S., Andreu-Sánchez, S., Vieira, F.G., Pedersen, C.B., Kinalis, S., Madsen, M.B., Kodama, M., Demircan, G.S., et al. (2021). A Comparison of Tools for Copy-Number Variation Detection in Germline Whole Exome and Whole Genome Sequencing Data. Cancers *13*, 6283.
11. Gordeeva, V., Sharova, E., Babalyan, K., Sultanov, R., Govorun, V.M., and Arapidi, G. (2021). Benchmarking germline CNV calling tools from exome sequencing data. Sci. Rep. *11*, 14416.
12. Samuels, D.C., Han, L., Li, J., Quanghu, S., Clark, T.A., Shyr, Y., and Guo, Y. (2013). Finding the lost treasures in exome sequencing data. Trends Genet. *29*, 593–599.
13. Royer-Bertrand, B., Castillo-Taucher, S., Moreno-Salinas, R., Cho, T.J., Chae, J.H., Choi, M., Kim, O.H., Dikoglu, E., Campos-Xavier, B., Girardi, E., et al. (2015). Mutations in the heat-shock protein A9 (HSPA9) gene cause the EVEN-PLUS syndrome of congenital malformations and skeletal dysplasia. Sci. Rep. *5*, 17154.
14. Wang, K., Li, M., and Hakonarson, H. (2010). ANNOVAR: functional annotation of genetic variants from high-throughput sequencing data. Nucleic Acids Res. *38*, e164.
15. Pedersen, B.S., and Quinlan, A.R. (2018). Mosdepth: quick coverage calculation for genomes and exomes. Bioinformatics *34*, 867–868.
16. Davis, T.L. (2022). optparse: Command Line Option Parser.
17. Warnes, G.R., Bolker, B., Bonebakker, L., Gentleman, R., Huber, W., Liaw, A., Lumley, T., Maechler, M., Magnusson, A., Moeller, S., et al. (2022). gplots: Various R Programming Tools for Plotting Data.
18. Plagnol, V., Curtis, J., Epstein, M., Mok, K.Y., Stebbings, E., Grigoriadou, S., Wood, N.W., Hambleton, S., Burns, S.O., Thrasher, A.J., et al. (2012). A robust model for read count data in exome sequencing experiments and implications for copy number variant calling. Bioinformatics *28*, 2747–2754.
19. Robin, X., Turck, N., Hainard, A., Tiberti, N., Lisacek, F., Sanchez, J.C., and Müller, M. (2011). pROC: an open-source package for R and S+ to analyze and compare ROC curves. BMC Bioinf. *12*, 77.
20. Tuszynski, J. (2021). caTools: Moving Window Statistics, GIF, Base64, ROC AUC, etc.
21. Geoffroy, V., Herenger, Y., Kress, A., Stoetzel, C., Piton, A., Dollfus, H., and Muller, J. (2018). AnnotSV: an integrated tool for structural variations annotation. Bioinformatics *34*, 3572–3574.
22. Robinson, J.T., Thorvaldsdóttir, H., Winckler, W., Guttman, M., Lander, E.S., Getz, G., and Mesirov, J.P. (2011). Integrative genomics viewer. Nat. Biotechnol. *29*, 24–26.
23. Mahamdallie, S., Ruark, E., Yost, S., Ramsay, E., Uddin, I., Wylie, H., Elliott, A., Strydom, A., Renwick, A., Seal, S., and Rahman, N. (2017). The ICR96 exon CNV validation series: a resource for orthogonal assessment of exon CNV calling in NGS data. Wellcome Open Res. *2*, 35.
24. Klambauer, G., Schwarzbauer, K., Mayr, A., Clevert, D.A., Mitterecker, A., Bodenhofer, U., and Hochreiter, S. (2012). cn.MOPS: mixture of Poissons for discovering copy number variations in next-generation sequencing data with a low false discovery rate. Nucleic Acids Res. *40*, e69.
25. Talevich, E., Shain, A.H., Botton, T., and Bastian, B.C. (2016). CNVkit: Genome-Wide Copy Number Detection and Visualization from Targeted DNA Sequencing. PLoS Comput. Biol. *12*, e1004873.

26. Jiang, Y., Wang, R., Urrutia, E., Anastopoulos, I.N., Nathanson, K.L., and Zhang, N.R. (2018). CODEX2: full-spectrum copy number variation detection by high-throughput DNA sequencing. Genome Biol. *19*, 202.
27. Krumm, N., Sudmant, P.H., Ko, A., O'Roak, B.J., Malig, M., Coe, B.P., NHLBI Exome Sequencing Project, Quinlan, A.R., Nickerson, D.A., and Eichler, E.E. (2012). Copy number variation detection and genotyping from exome sequence data. Genome Res. *22*, 1525–1532.
28. Boeva, V., Popova, T., Bleakley, K., Chiche, P., Cappo, J., Schleiermacher, G., Janoueix-Lerosey, I., Delattre, O., and Barillot, E. (2012). Control-FREEC: a tool for assessing copy number and allelic content using next-generation sequencing data. Bioinformatics *28*, 423–425.
29. McKenna, A., Hanna, M., Banks, E., Sivachenko, A., Cibulskis, K., Kernytsky, A., Garimella, K., Altshuler, D., Gabriel, S., Daly, M., and DePristo, M.A. (2010). The Genome Analysis Toolkit: a MapReduce framework for analyzing next-generation DNA sequencing data. Genome Res. *20*, 1297–1303.
30. Laver, T.W., De Franco, E., Johnson, M.B., Patel, K.A., Ellard, S., Weedon, M.N., Flanagan, S.E., and Wakeling, M.N. (2022). SavvyCNV: Genome-wide CNV calling from off-target reads. PLoS Comput. Biol. *18*, e1009940.
31. Henderson, R.H. (2020). Inherited retinal dystrophies. Paediatr. Child Health *30*, 19–27.
32. Richards, S., Aziz, N., Bale, S., Bick, D., Das, S., Gastier-Foster, J., Grody, W.W., Hegde, M., Lyon, E., Spector, E., et al. (2015). Standards and guidelines for the interpretation of sequence variants: a joint consensus recommendation of the American College of Medical Genetics and Genomics and the Association for Molecular Pathology. Genet. Med. *17*, 405–424.
33. Riggs, E.R., Andersen, E.F., Cherry, A.M., Kantarci, S., Kearney, H., Patel, A., Raca, G., Ritter, D.I., South, S.T., Thorland, E.C., et al. (2020). Technical standards for the interpretation and reporting of constitutional copy-number variants: a joint consensus recommendation of the American College of Medical Genetics and Genomics (ACMG) and the Clinical Genome Resource (ClinGen). Genet. Med. *22*, 245–257.
34. Nikopoulos, K., Cisarova, K., Quinodoz, M., Koskiniemi-Kuendig, H., Miyake, N., Farinelli, P., Rehman, A.U., Khan, M.I., Prunotto, A., Akiyama, M., et al. (2019). A frequent variant in the Japanese population determines quasi-Mendelian inheritance of rare retinal ciliopathy. Nat. Commun. *10*, 2884.
35. Torene, R.I., Galens, K., Liu, S., Arvai, K., Borroto, C., Scuffins, J., Zhang, Z., Friedman, B., Sroka, H., Heeley, J., et al. (2020). Mobile element insertion detection in 89,874 clinical exomes. Genet. Med. *22*, 974–978.
36. Bellos, E., and Coin, L.J.M. (2014). cnvOffSeq: detecting intergenic copy number variation using off-target exome sequencing data. Bioinformatics *30*, i639–i645.
37. Kuilman, T., Velds, A., Kemper, K., Ranzani, M., Bombardelli, L., Hoogstraat, M., Nevedomskaya, E., Xu, G., de Ruiter, J., Lolkema, M.P., et al. (2015). CopywriteR: DNA copy number detection from off-target sequence data. Genome Biol. *16*, 49.

The American Journal of Human Genetics, Volume *111*

Supplemental information

Detection of elusive DNA copy-number variations in hereditary disease and cancer through the use of noncoding and off-target sequencing reads

Mathieu Quinodoz, Karolina Kaminska, Francesca Cancellieri, Ji Hoon Han, Virginie G. Peter, Elifnaz Celik, Lucas Janeschitz-Kriegl, Nils Schärer, Daniela Hauenstein, Bence György, Giacomo Calzetti, Vincent Hahaut, Sónia Custódio, Ana Cristina Sousa, Yuko Wada, Yusuke Murakami, Almudena Avila Fernández, Cristina Rodilla Hernández, Pablo Minguez, Carmen Ayuso, Koji M. Nishiguchi, Cristina Santos, Luisa Coutinho Santos, Viet H. Tran, Veronika Vaclavik, Hendrik P.N. Scholl, and Carlo Rivolta

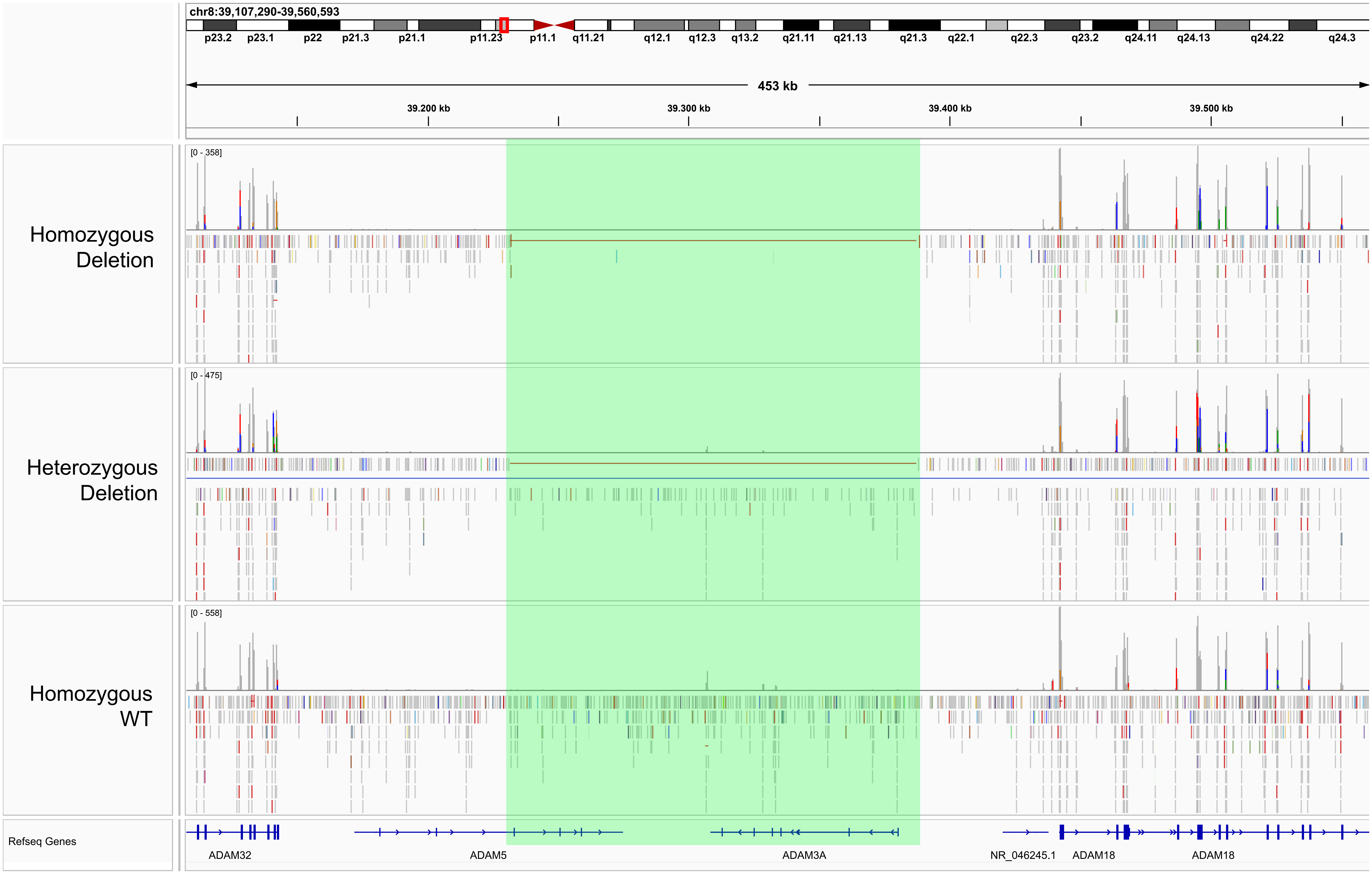

Figure S1: IGV view of a typical deletion involving mostly off-target regions. A common deletion on chromosome 8 is shown (gnomAD: DEL_8_91589, in green). Sequencing data presented here are derived from (top to bottom): a homozygous, a heterozygous, and a wild type (WT) individual for the deletion. Split reads are shown as red horizontal lines.

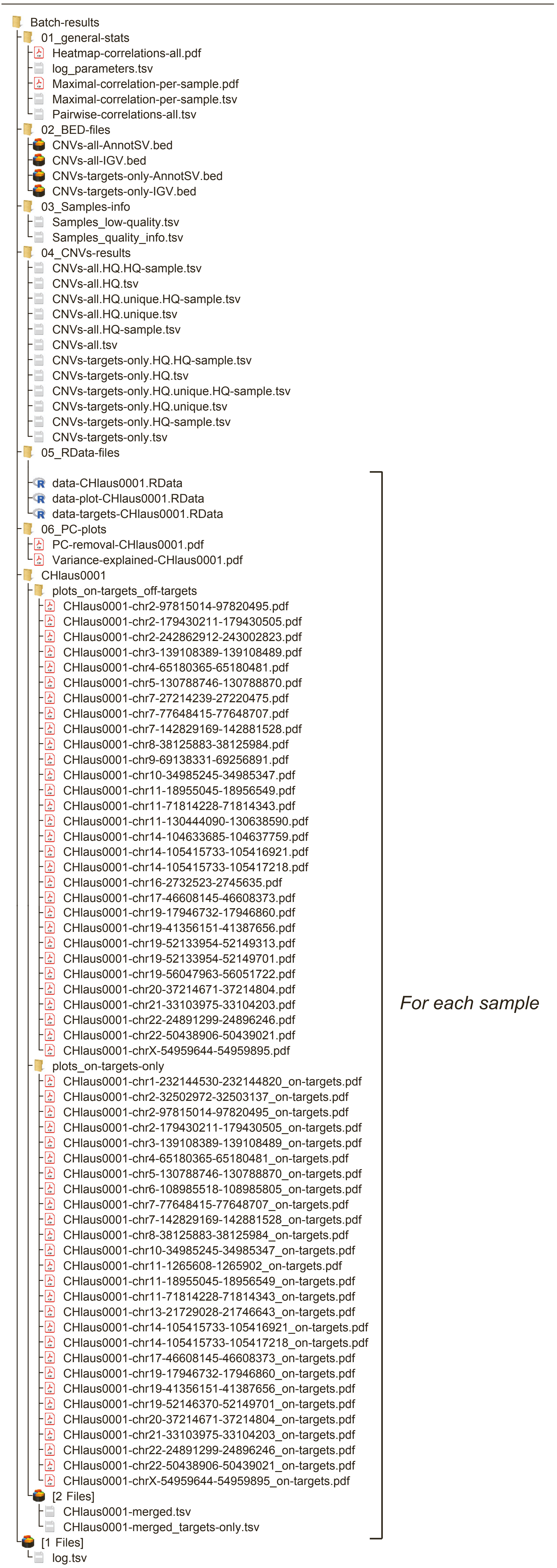

Figure S2: Typical output files of OFF-PEAK, here for individual CHlaus0001. The files listed at the top are common to all samples belonging to the same batch, whereas those listed at the bottom are specific for each sample.

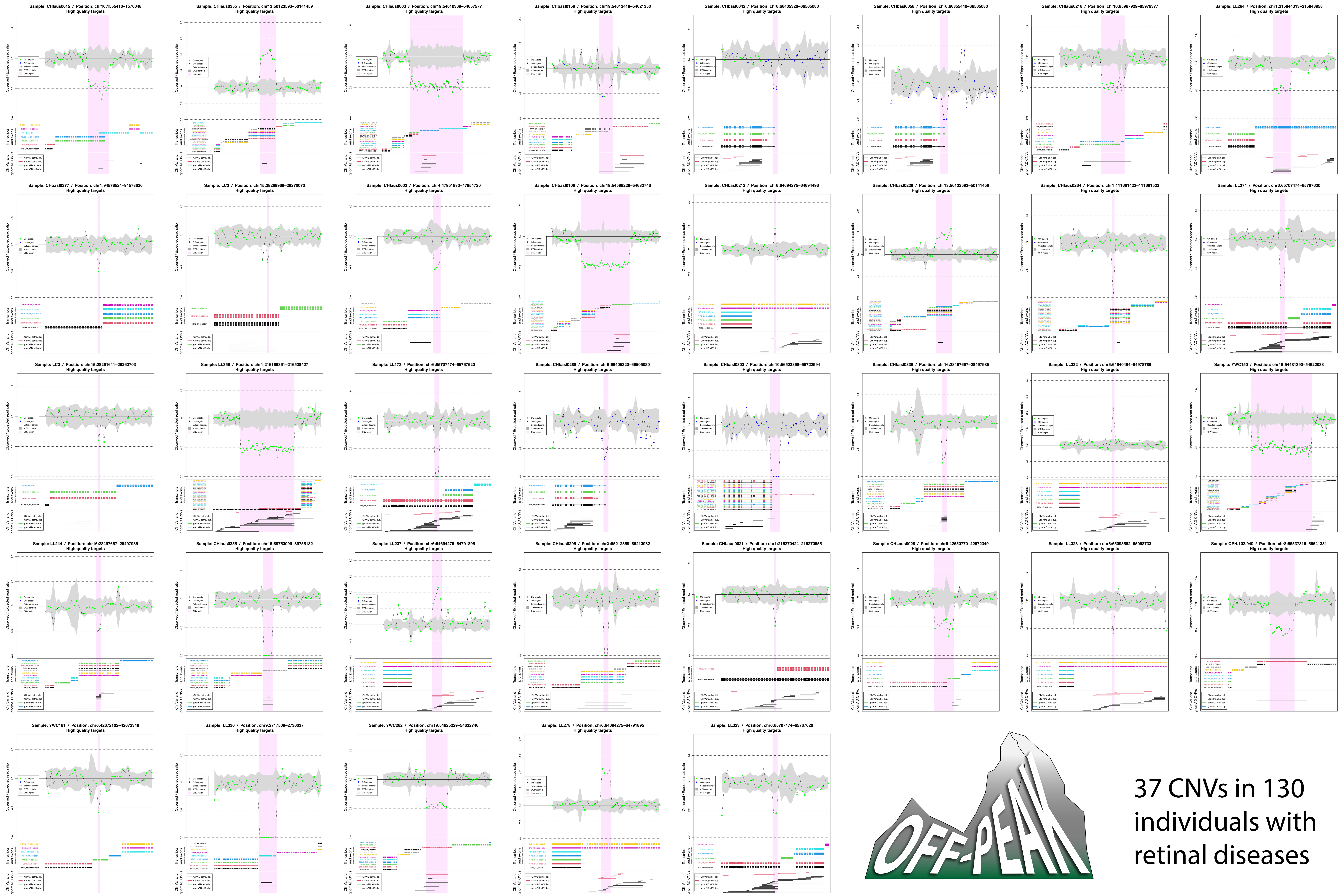

Figure S3: OFF-PEAK graphical outputs for all validated CNVs, detected in the retinal diseases cohort.

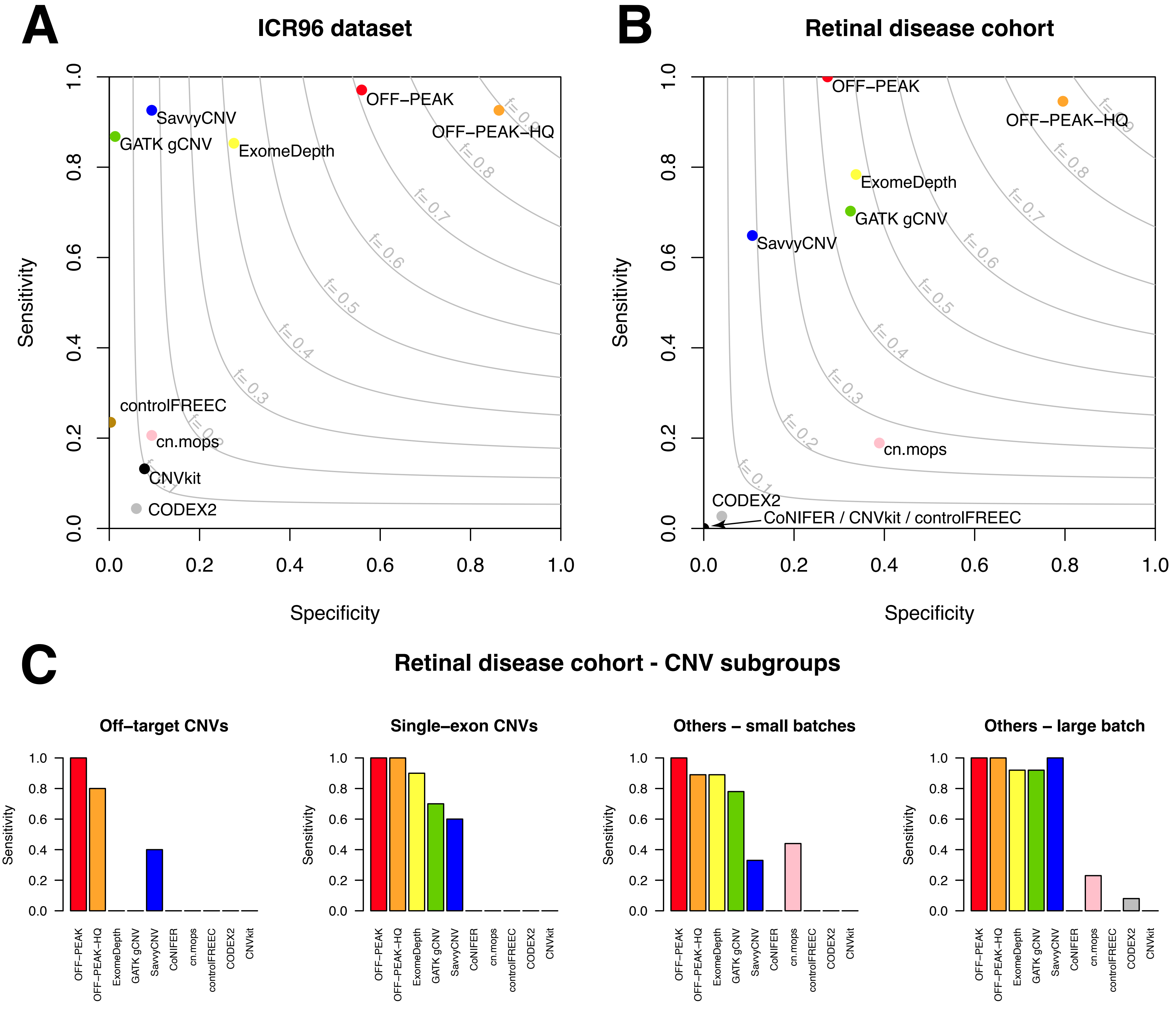

Figure S4: Performance of OFF-PEAK and other tools with respect to different testing sets, taking only CNVs with exact captured regions detected. (A) Specificity-sensitivity plot for the ICR96 dataset of 96 cancer samples, on 68 validated CNVs. (B) Specificity-sensitivity plot for the cohort of 130 individuals with retinal phenotypes, on 37 validated CNVs. (C) Bar plots of sensitivities for the latter cohort, stratified according to the type of CNVs considered: off-target (e.g. noncoding), on-target single-exon, small batches (kits 1 and 3), and large batch (kit 2, see Sup. Methods). The curves in light grey represent the F-score or harmonic mean of sensitivity and specificity.

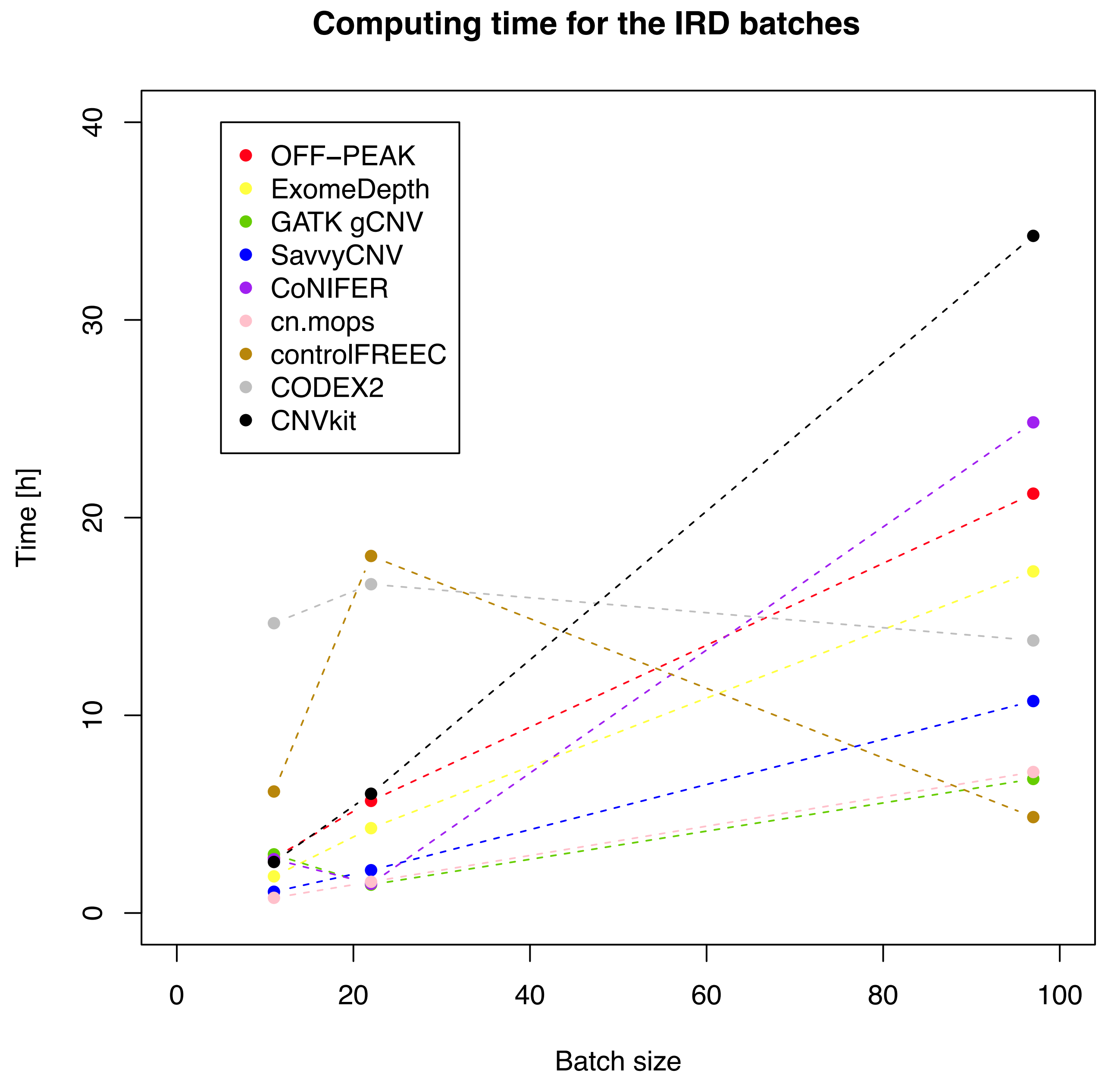

Figure S5: Computing time for three WES batches of various sizes (kit 1, n =11; kit 2, n=97; kit 3, n = 22).

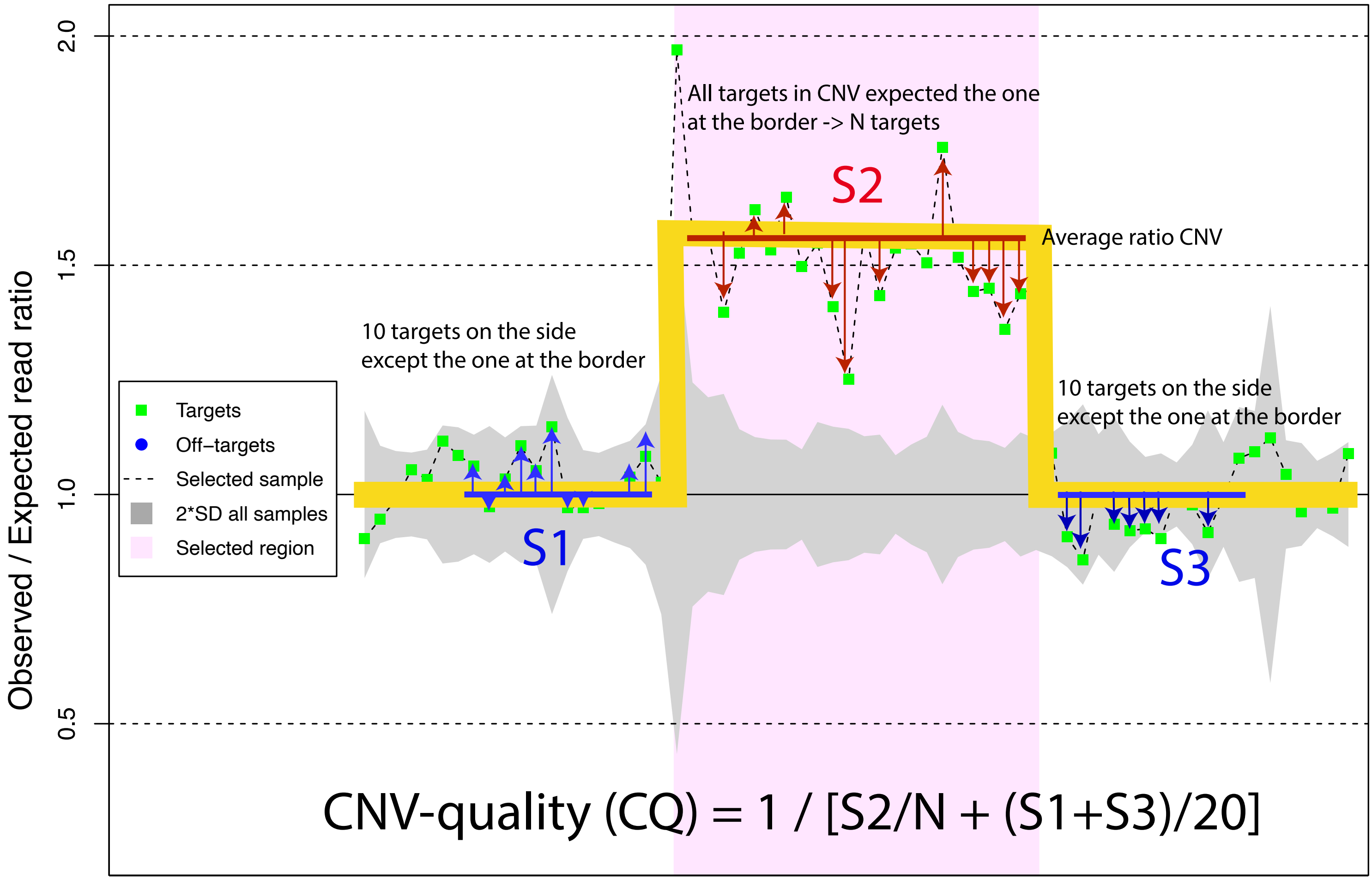

Figure S6: Schematic view of CNV-quality (CQ) computation. A CNV determines an abrupt change in coverage, which can be represented by a rectangular function (in yellow). We can estimate the quality of a CNV by computing how close such variation of coverage and this rectangular function are. For this purpose, we sum the deviations in coverage of the targets (green squares) from this function. Deviations occurring outside of the CNV are highlighted by blue arrows, whereas red arrows highlight those occurring within the CNV. The exact equation estimating CNV quality is indicated at the bottom of the image.

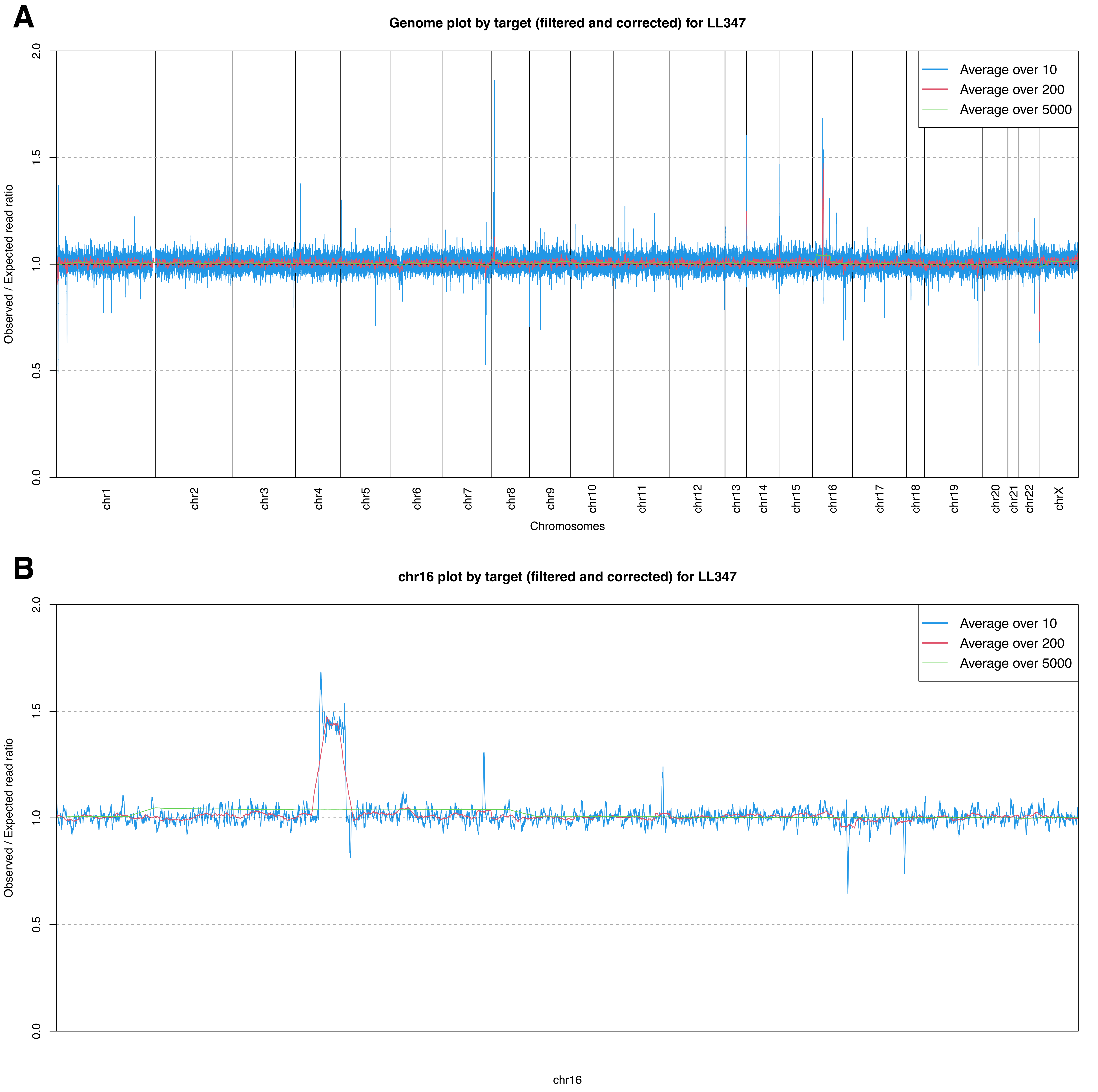

Figure S7: Examples of a genome-wide coverage plot (A) and a chromosome-specific coverage plot (B) for individual LL347, who carries a large heterozygous duplication on chromosome 16.

Supplementary Methods

Details about noise removal using LOO-PCA

Noise removal was done using a leave-one-out principal components analysis (LOO-PCA) approach. Specifically, the optimized number of PCs to be removed was computed by:

1. Downsampling of the targets, by randomly selecting a specific number of them (--downsample, default: 20,000).
2. Creation of artificial (fake) heterozygous deletions and duplications by halving or multiplying by 1.5 times the real coverage value of a defined number of regions in the test sample (--nbFake, default: 500 each).
3. Computing PCA on the control samples only (LOO-PCA).
4. Starting to recover CNVs without PC removal, then incrementing the removal of PCs and computing at every step the AUC based on Z-scores of the test sample compared to control samples. The optimized PC removal was defined when the performance increased by less than a specific threshold (--stopPC, default: 0.0001). If the threshold was passed, 10 more PCs were sequentially removed to try to increase the performance.

CNV annotation and graphical representation by OFF-PEAK

Following the detection process, all CNVs were annotated to include: the sample they belonged to, their genomic coordinates (minimal and maximal), their type (deletion or duplication), their reads ratio compared to controls, Z-score, ploidy, affected targets, affected

exons, genes, non-coding RNAs and functional elements affected (RefSeq), the number of samples with overlapping CNVs, the overlapping CNVs from ClinVar and gnomAD databases, as well as with various quality metrics.

The quality metrics were:

- PQ (ploidy quality) = minimal difference of Z-score between the called ploidy and other ploidies divided by number of targets
- CQ (CNV quality) = measure of CNV quality based on divergence from a rectangular function (difference between expected and observed ratios), defined as:

$$CQ = \frac{1}{\frac{S2}{N} + \frac{S1+S3}{20}}$$

for a CNV going from target *k* to *m*:

$$S1 = abs\left[\sum_{k-11}^{k-2} (x_i - 1)\right]$$

$$S2 = abs\left[\sum_{k+1}^{m-1} \left(x_i - \frac{\sum_{i=k+1}^{m-1} x_i}{m-k-2}\right)\right]$$

$$S3 = abs\left[\sum_{m+2}^{m+11} (x_i - 1)\right]$$

(see Figure S6)

- QUAL = Z-score * PQ * CQ / N-targets. It measures both, the quality of the called ploidy (PQ), and that of the correct CNV "shape" (CQ)

The graphical representation of every CNV detected shows the ratio between the sample considered and the control samples, the genes and exons involved, as well as frequent CNVs

from gnomAD and pathogenic CNVs from ClinVar. The canonical transcripts were indicated based on NCBI RefSeq Select.

Other output files by OFF-PEAK

These additional output files were created when running OFF-PEAK:

- Detected CNVs with annotations
- CNV plots for top 20 CNVs per sample, including CNVs found in ClinVar and gnomAD databases (examples in Figure 5 and Figure S3)
- Heatmap of the correlation between samples and the table of pairwise correlations
- Bar chart of the maximal pairwise correlation per sample, as well as the data in text format
- BED file compatible with the AnnotSV software[1]
- BED file for the Integrative Genomics Viewer[2]
- Sample information, such as quality or number of detected CNVs
- RData files for creating additional plots
- Plot showing performance of PC removal on artificial CNVs and plot showing the cumulative explained variance
- Genome and chromosome plots (example in Figure S7)

Parameters used for CNV detection on the ICR96 data

ICR96 data (FASTQ files and target BED file)[3] were downloaded from the European Genome-phenome Archive (EGA), with the agreement of Prof. Nazneen Rahman at The

Institute of Cancer Research, London. The FASTQ files were used as described in the "Mapping and variant calling" section to produce processed BAM files. CNV detection was run as follows:

- OFF-PEAK: default parameters with target BED file
- cn.mops: default parameters
- CNVkit: default parameters with target BED file
- CODEX2: default parameters with target BED file, without chromosome X
- CoNIFER: did not work due to insufficient number of target regions ("Error: This chromosome has fewer informative probes than there are samples in the analysis!")
- ExomeDepth: default parameters
- Control-FREEC: default parameters
- GATK: default parameters with target BED file
- SavvyCNV: default parameters; after running CoverageOffTarget command, SavvyCNV was run with -d 200

Data processing for the validation on the ICR96 dataset

We retrieved the outputs of each tool and processed them as follow:

- OFF-PEAK: all CNVs detected (deletion if ratio < 1 and duplication if ratio > 1) affecting targets (CNVs-targets-only.tsv file)
- OFF-PEAK-HQ: all HQ CNVs detected (deletion if ratio < 1 and duplication if ratio > 1) affecting targets (CNVs-targets-only.HQ.tsv file)
- cn.mops all CNVs detected (deletion if CN < 2 and duplication if CN > 2)

- CNVkit: all CNVs detected (deletion if CN < 2 and duplication if CN > 2)
- CODEX2: all CNVs detected (deletion if lratio < 2 and duplication if lratio > 2)
- CoNIFER: the tool could not be run on this dataset
- ExomeDepth: all CNVs detected (deletion if Reads-ratio < 1 and duplication if Reads-ratio > 1)
- Control-FREEC: all CNVs detected (deletion if CN < 2 and duplication if CN > 2)
- GATK: all CNVs detected (deletion if CN < 2 and duplication if CN > 2)
- SavvyCNV: all CNVs detected (deletion if relative dosage < 1 and duplication if relative dosage > 1)

All detected CNVs overlapping with the true set and with the correct ploidy (heterozygous or homozygous events) were considered as true positives. Events without any overlaps were considered as false negatives. In a second step, the same analysis was repeated for CNVs overlapping exactly the captured regions found in the validated CNVs. Sensitivity was computed as the number of detected CNVs divided by the total number of validated CNVs; specificity as the number of validated CNVs divided by the total number of detected CNVs.

Parameters used for CNV detection in the retinal disease samples

CNV detection was run separately for each capture kit (1 to 3), as follows:

- OFF-PEAK: default parameters with target BED file, using both, all-targets, and on-targets-only output. Columns “begin-min” and “end-max” were used for additional analysis. For performance analysis, overlapping CNVs detected by on-target and off-target approaches were counted only once. For further investigation with lower

stringency, the following parameters were used: minOntarget=30, maxOntarget=75, minZ=3.

- cn.mops: default parameters
- CNVkit: default parameters with target BED file
- CODEX2: default parameters with target BED file, without chromosome X
- CoNIFER: default parameters with target BED file, with 1 SVD component removed for kit 1 and kit 3. For kit 2, CoNIFER BED file was used with 4 SVD components removed
- ExomeDepth: default parameters
- Control-FREEC: default parameters
- GATK: default parameters with target BED file
- SavvyCNV: default parameters; after running CoverageOffTarget command, SavvyCNV was run with -d 200 for on-target analysis; for off-target analysis, it was run with -d 19800 for kit 1, -d 29800 for kit 2, and -d 27400 for kit 3. For performance analysis, overlapping CNVs detected by on-target and off-target approaches were counted only once.

The computing time for each batch and each tool was recorded. All tools were used on the same machine, a DELL Power Edge R640 with 36 CPUs (Intel Xeon Gold 6150).

SNV filtering for the retinal disease cohort

DNA variants were filtered to be rare, with an allelic frequency lower than 0.01 in gnomAD,[4] as well as in ToMMo,[5] ABraOM,[6] ESP6500,[7] and present in an in-house inventory of sequenced samples. Moreover, they were selected to be of high quality (GQ > 30,

allelic_ratio > 0.25, FS < 25, VQSLOD > -5, and ExcessHet < 50) and to have a predicted impact at the protein level (nonsense, frameshift, missense, canonical splice site or splicing variants [MaxEntScan, SpliceAI and dbscSNV-ADA]). Variants were further selected to affect genes known to cause IRD phenotypes based on OMIM[8] (Table S4).

Detection of potentially pathogenic CNVs in the IRD cohort

The CNVs detected by all tools except controlFREEC (due to too many calls, low specificity) and CNVkit (too many CNVs called for a few specific cases) were first selected to be rare (detected in less than 5 samples per tool) and to affect genes known to cause retinal phenotypes. During the selection process the operator was not blinded with respect to the tool examined, but there was no bias in the selection of calls, which were all considered as equivalent and processed in the same way in the following steps. All genes were first selected from OMIM to have a solid association with IRDs and then filtered to have known mechanism of disease corresponding to loss-of-function (for autosomal recessive and X-linked conditions) or haploinsufficiency (for autosomal dominant phenotypes) (Table S4).

In addition, CNVs linked to autosomal recessive or X-linked phenotypes were retained if:

- they were detected as likely homozygous events (homozygous deletion or duplication defined as ploidy < 0.25 (for deletions), or between 3.25 and 5 (for duplications)
- there were two CNVs detected in the same gene (compound heterozygous)
- there was a rare and deleterious SNV or small indel affecting the same genes (compound heterozygous)

and for autosomal dominant phenotypes if:

- they were detected as likely heterozygous events (ploidy is not 2)

The selected events were then manually filtered to make sure they were: not artefactual (based on IGV visualization of BAM files), affecting exonic regions, and compatible with the phenotype of all affected individuals (Table S3). They were then all validated either through PCR, WGS or MLPA (Table 1). The list of primers used for the PCR validations can be found in Table S11. MLPA analysis for copy number variation detection on *EYS* gene was carried out using MLPA Salsa P328-A2 probemix (MRC Holland, Netherlands). WGS was performed either at CeGaT GmbH (Tübingen, Germany) or at D-BSSE (ETHZ, Basel) on a Novaseq 6000 (CeGaT) or a Novaseq SP flowcell (50-8-8-50, D-BSSE). Libraries were generated with TruSeq DNA PCR-Free kit (Illumina) for CeGaT or with the Watchmaker DNA Library Prep Kit with Fragmentation (Watchmaker Genomics, USA) and indexed with TruSeq™–Compatible Duplex Y Adapters (Integrated DNA Technology) for D-BSSE.

Data processing for the validation on the IRD set

All detected CNVs overlapping with the true set and with the correct ploidy (heterozygous or homozygous events) were considered as true positives. Events without any overlaps were considered as false negatives. In a second step, the same analysis was repeated for CNVs overlapping exactly the captured regions found in the validated CNVs. Sensitivity was computed as the number of detected CNVs divided by the total number of validated CNVs; specificity as the number of validated CNVs divided by the total number of detected CNVs. To avoid double scoring of the same events, the total number of CNVs detected by OFF-PEAK was the sum of non-overlapping CNVs for all targets and of on-target-only analyses. Similarly,

for SavvyCNV, the total number of CNVs was the sum of non-overlapping CNVs from on-target and off-target analyses.

References

1. Geoffroy, V., Herenger, Y., Kress, A., Stoetzel, C., Piton, A., Dollfus, H., and Muller, J. (2018). AnnotSV: an integrated tool for structural variations annotation. Bioinformatics 34, 3572-3574.

2. Robinson, J.T., Thorvaldsdottir, H., Winckler, W., Guttman, M., Lander, E.S., Getz, G., and Mesirov, J.P. (2011). Integrative genomics viewer. Nat Biotechnol 29, 24-26.

3. Mahamdallie, S., Ruark, E., Yost, S., Ramsay, E., Uddin, I., Wylie, H., Elliott, A., Strydom, A., Renwick, A., Seal, S., et al. (2017). The ICR96 exon CNV validation series: a resource for orthogonal assessment of exon CNV calling in NGS data. Wellcome Open Res 2, 35.

4. Karczewski, K.J., Francioli, L.C., Tiao, G., Cummings, B.B., Alfoldi, J., Wang, Q., Collins, R.L., Laricchia, K.M., Ganna, A., Birnbaum, D.P., et al. (2020). The mutational constraint spectrum quantified from variation in 141,456 humans. Nature 581, 434-443.

5. Ogishima, S., Nagaie, S., Mizuno, S., Ishiwata, R., Iida, K., Shimokawa, K., Takai-Igarashi, T., Nakamura, N., Nagase, S., Nakamura, T., et al. (2021). dbTMM: an integrated database of large-scale cohort, genome and clinical data for the Tohoku Medical Megabank Project. Hum Genome Var 8, 44.

6. Naslavsky, M.S., Scliar, M.O., Yamamoto, G.L., Wang, J.Y.T., Zverinova, S., Karp, T., Nunes, K., Ceroni, J.R.M., de Carvalho, D.L., da Silva Simoes, C.E., et al. (2022). Whole-genome sequencing of 1,171 elderly admixed individuals from Sao Paulo, Brazil. Nat Commun 13, 1004.

7. Fu, W., O'Connor, T.D., Jun, G., Kang, H.M., Abecasis, G., Leal, S.M., Gabriel, S., Rieder, M.J., Altshuler, D., Shendure, J., et al. (2013). Analysis of 6,515 exomes reveals the recent origin of most human protein-coding variants. Nature 493, 216-220.

8. Amberger, J.S., Bocchini, C.A., Schiettecatte, F., Scott, A.F., and Hamosh, A. (2015). OMIM.org: Online Mendelian Inheritance in Man (OMIM(R)), an online catalog of human genes and genetic disorders. Nucleic Acids Res 43, D789-798.
